# X-ray synchrotron radiation studies of actinide materials

**DOI:** 10.1107/S1600577521009413

**Published:** 2021-11-01

**Authors:** Roberto Caciuffo, Gerard H. Lander

**Affiliations:** a European Commission, Joint Research Centre, Postfach 2340, D-76125 Karlsruhe, Germany; b Interface Analysis Centre, School of Physics, University of Bristol, Tyndall Avenue, Bristol BS8 1TL, United Kingdom

**Keywords:** actinides, hidden order, multipoles, phonons, electronic structure

## Abstract

By reviewing a selection of X-ray diffraction (XRD), resonant X-ray scattering (RXS), X-ray magnetic circular dichroism (XMCD), resonant and non-resonant inelastic scattering (RIXS, NIXS), and dispersive inelastic scattering (IXS) experiments, the potential of synchrotron radiation techniques in studying lattice and electronic structure, hybridization effects, multipolar order and lattice dynamics in actinide materials is demonstrated.

## Abstract 2

Synchrotron radiation based X-ray scattering techniques provide powerful bulk-sensitive probes for actinide materials. Here, selected experiments showing the potential of these techniques in advancing the knowledge of actinides and their compounds are reviewed.

## 1. Introduction

Actinides are the heaviest chemical elements available on a macroscopic scale. The complexity of their electronic structure often produces exotic physical properties, such as the unconventional superconductivity of PuCoGa_5_ and the hidden-order phases found in NpO_2_ and URu_2_Si_2_ (Sarrao *et al.*, 2002 ▸; Mydosh & Oppeneer, 2011 ▸; Moore & van der Laan, 2009 ▸; Santini *et al.*, 2009 ▸). The richness of actinide physics has multiple origins. Intra-atomic Coulomb correlations compete with spin–orbit, exchange, and crystal field interactions on a comparable energy scale; scalar relativistic effects influence the spatial extension of the 5*f* electron shell; hybridization between 5*f* and conduction electron states is strong, attributing to the 5*f* electrons a dual localized-itinerant character.

X-ray synchrotron radiation techniques provide powerful tools to unravel the complexity of actinide materials. These elements- and shell-specific techniques probe spatial and temporal fluctuations of structural and electronic degrees of freedom, allowing one to observe hidden-order parameters and characterize elementary excitations with high sensitivity and resolution. Contrary to neutron scattering, synchrotron radiation experiments require samples on the microgram scale. This is important for actinides, as large quantities are difficult to manipulate and large single crystals are rare.

**Figure 1:**
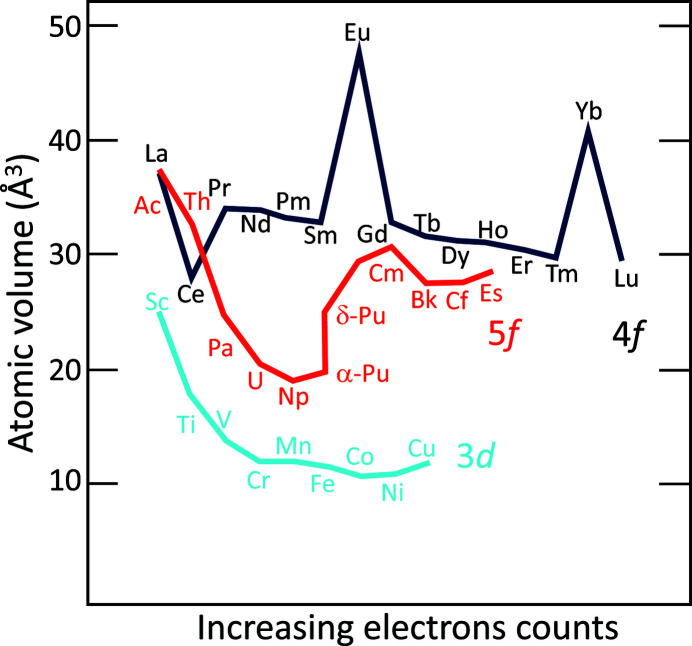
Atomic volume of the transition (3*d*), rare-earth (4*f*), and actinide (5*f*) elements as a function of electron count. Note the unusual shape of the curve for the actinide series. In the case of Eu and Yb the large expansion is due to these elements being stable in the divalent state. All other rare-earth elements have the trivalent ground state.

A central feature of the research on actinides can be illustrated by Fig. 1 ▸ and the atomic volume as a function of electron count across the 3*d*, 4*f*, and 5*f* series of elements. For the 3*d* elements, additional 3*d* electrons result in a *contraction* of the atomic volume as each additional electron adds to the cohesion of the element, resulting in a smaller atomic volume. In the 4*f* (rare-earth) series, apart from the two divalent elements Eu and Yb, the atomic volume is practically constant across the series. This is because the 4*f* electrons are spatially located close to the nucleus and are not involved in the bonding. However, for the 5*f* (actinide) series both behaviors are observed: an initial drop in the volume up to α-Pu, suggesting the 5*f* states are contributing to the bonding and are therefore *itinerant* for the light actinides, and then a strong expansion for other phases of Pu and through to heavier actinide elements, hence suggesting a *localization* of the 5*f* states from Am onwards.

Actinide elements, unlike most metals, crystallize in open-packed, low-symmetry structures. The behavior of elemental plutonium, exhibiting six ambient pressure allotropes, is exemplary (Zachariasen & Ellinger, 1955 ▸, 1957 ▸, 1963*a* ▸,*b* ▸; Lashley & Lawson, 2019 ▸). Its large volume expansion is unique in the actinides, and is particularly dramatic. As Pu is heated, its density decreases by about 20% from the room-temperature α-Pu form (simple monoclinic, space group *P*2_1_/*m*) until reaching the high-symmetry face-centered cubic δ phase ( 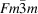 ), stable between 315 and 452°C. Then, plutonium contracts with a density increase of ∼4% while transforming to the body-centered cubic ε phase that eventually contracts on melting at 639.4°C. One obvious experiment is to compress Am and heavier actinides and see whether the resulting compressed elements have the open crystal structures found for the more itinerant lighter actinide elements.

## 2. X-ray diffraction

X-ray diffraction is certainly the technique of choice for determining crystallographic structures. Most of the ordinary crystallography is, however, carried out with conventional X-ray sources, and this is particularly true for actinides due to safety requirements. Synchrotron radiation facilities become necessary for high resolution or high pressure studies. The latter, in particular, have played an important role in the development of first-principles electronic structure calculations methods.

Electrons in the 5*f* shell are prone to an instability between localized and magnetic states to itinerant and bonding ones. Although metallic, elemental light actinides exhibit low-symmetry open-packed structures, which is unusual for simple metals. Moreover, a pressure of the order of a few GPa is sufficient to destabilize their crystallographic structure and induce a sequence of phase transformations. In some cases, the structural changes are accompanied by volume collapses indicating a stepwise delocalization of 5*f* electrons and their increasing contribution to the crystal cohesion energy.

**Figure 2:**
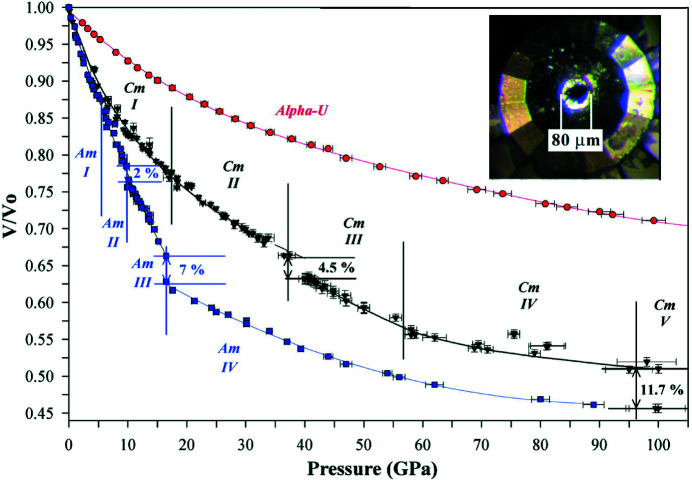
Pressure dependence of the normalized unit cell volume, *V*(*p*)/*V*_0_, for α-uranium (Le Bihan *et al.*, 2003 ▸), americium (Lindbaum *et al.*, 2001 ▸), and curium (Heathman *et al.*, 2005 ▸). The inset shows a typical diamond anvil cell used for angle-dispersive, high-pressure X-ray diffraction experiments. Adapted from Heathman *et al.* (2005 ▸). Reprinted with permission from AAAS.

Some examples are shown in Fig. 2 ▸ showing the equation of states for α-U, Am, and Cm as determined by high-pressure X-ray diffraction. Under compression, uranium preserves its room-temperature-stable orthorhombic *Cmcm* form up to at least 100 GPa. A different behavior is observed for americium where the normal pressure double-hexagonal close-packed (*P*6_3_/*mmc*) structure transforms at 6.1 GPa to a face centered cubic ( 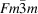 ) lattice. At higher pressures, two lower symmetry structures appear, a face-centered orthorhombic Am III (*Fddd*) and a primitive orthorhombic structure, Am IV (*Pnma*). In the same pressure range, up to 100 GPa, curium exhibits five phases, from the double-hexagonal close-packed (d.h.c.p.) form of Cm I (*P*6_3_/*mmc*) to the ortho­rhombic (*Pnma*) structure of Cm V. Of particular interest is the formation of the monoclinic structure with the space group *C*2/*c* between about 37 and 56 GPa. Calculations based on the full potential linear muffin-tin orbital (FPLMTO) method suggest that its stabilization is driven by the magnetic correlation energy (Heathman *et al.*, 2005 ▸). The collapse of Am and Cm from simple to complex structures under pressure shows, in keeping with Fig. 1 ▸, that the 5*f* electrons have transformed from localized to itinerant under pressure.

Beyond Cm, the available quantities become very small, but recent work has shown that the concept of de-localization under pressure may be too simple a picture. Examples are pressure work on Cf (Heathman *et al.*, 2013 ▸) and spectroscopy work on Es (Carter *et al.*, 2021 ▸), both using synchrotron radiation techniques.

Reproducing from first-principles electronic structure calculations the observed sequence of lattice geometry and the associated evolution of physical properties in actinide elements and compounds is challenging. Simple approximations of the density functional theory, in fact, are not adequate for actinide materials, because of the strong intra-atomic correlations, the importance of scalar (first-order kinetic energy correction due to the mass variation) and non-scalar (spin–orbit coupling) relativistic effects, and the extent of the hybridization between 5*f* and conduction electrons. In many cases a model that produces the correct crystal structure at a certain atomic volume fails to describe the electronic structure near the Fermi level, and does not reproduce the correct magnetic state. This is the case, for instance, of conventional density functional theory (DFT) calculations in the local-spin density or generalized-gradient approximation (LDA/GGA) applied to δ-Pu (Lashley *et al.*, 2005 ▸; Joyce & Lander, 2019 ▸), or of static mean field correlated band theory calculations making use of different flavors of the LDA/GGA plus Coulomb’s U (LDA + U) method, falling short in describing the itinerant-to-localized crossover of the 5*f* manifold in PuCoGa_5_ (Daghero *et al.*, 2012 ▸; Shick *et al.*, 2013 ▸). During the last two decades, experiments such as those reported in Fig. 2 ▸ have, therefore, stimulated the development of increasingly sophisticated theoretical models that have now reached predicting capabilities close to a material-by-design level also for compounds in the 5*f* block (Shim *et al.*, 2007 ▸; Suzuki *et al.*, 2010 ▸; Pezzoli *et al.*, 2011 ▸; Dudarev *et al.*, 2019 ▸; Pourovskii & Khmelevskyi, 2021 ▸; Shick *et al.*, 2021 ▸).

**Figure 3:**
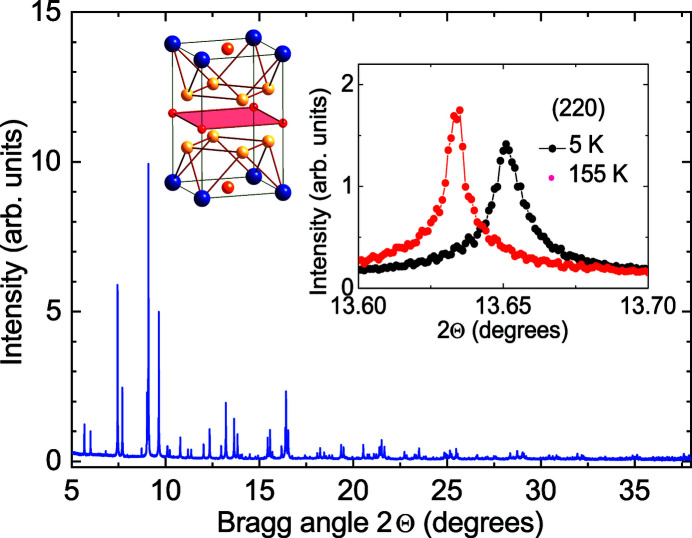
X-ray diffraction pattern recorded for PuCoGa_5_ at 5 K. The inset on the left shows the tetragonal crystallographic unit cell, with Pu and Co atoms represented by blue and red spheres, respectively, whilst the two inequivalent Ga atoms are shown by orange and yellow spheres. The inset on the right shows the (2 2 0) Bragg peak measured at 5 K (black circles) and 21 K (red circles). Reprinted with permission from Eloirdi *et al.* (2017 ▸). Copyright (2017) by the American Physical Society.

As an example of high resolution XRD study, we show in Fig. 3 ▸ the results obtained for the PuCoGa_5_ unconventional superconductor (Eloirdi *et al.*, 2017 ▸). The experiment was performed at the ESRF ID22 beamline, affording a resolution Δ*d*/*d* = 10^−6^ at λ = 0.354155 Å. Data were collected on a 4.6 mg sample, obtained by crushing a single crystal grown from metallic plutonium (99.932 wt% ^242^Pu), put inside a hermetic holder providing four levels of containment. The absence of visible splitting or broadening of the diffraction peaks, as seen in the inset of Fig. 3 ▸ for the (2 2 0) Bragg reflection, indicates that the tetragonal symmetry is preserved in the superconducting phase. The temperature dependence of the refined lattice parameters shows that the thermal expansion is isotropic above ∼150 K. At lower temperatures, the *c*/*a* ratio increases with decreasing *T*. In the same temperature range, Ramshaw *et al.* (2015 ▸) observe a softening of the bulk modulus that they attribute to the development of in-plane hybridization between conduction electron and Pu 5*f*.

**Figure 4:**
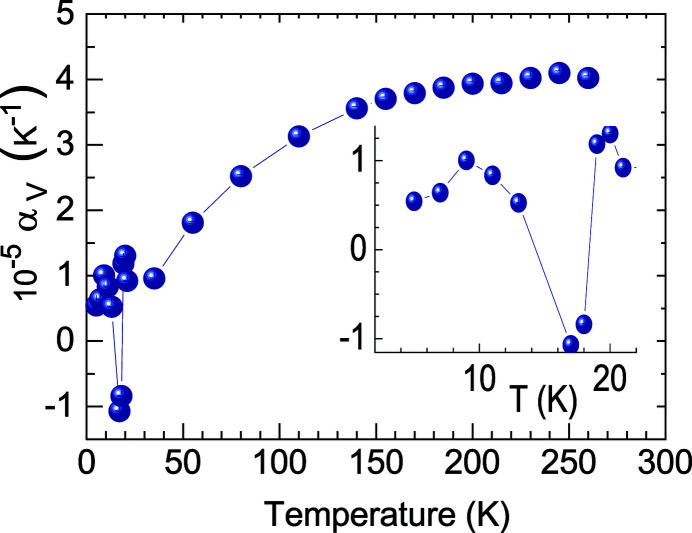
Thermal expansion coefficient for the unit cell volume of PuCoGa_5_ (in an expanded scale around *T*_c_ in the inset). Error bars have been estimated as five times the statistical error provided by the Rietveld refinement. If not shown, they are smaller than the data symbol size. Reprinted with permission from Eloirdi *et al.* (2017 ▸). Copyright (2017) by the American Physical Society.

Below *T*_c_ = 18.5 K, the critical temperature to the superconducting state in PuCoGa_5_, the expansion of the unit cell volume deviates from the predictions of a simple one-phonon Grüneisen–Einstein model (Fig. 4 ▸). The shrinking of the cell volume is similar to the one observed for the CeRu_2_Si_2_ Kondo system (Hiranaka *et al.*, 2013 ▸) and could suggest the occurrence of critical valence fluctuations at *T*_c_ (Miyake & Watanabe, 2014 ▸), where the volume thermal expansion coefficient α_V_ has a jump larger by a factor of ∼20 than the value predicted by the Ehrenfest relation.

## 3. Resonant X-ray scattering

Resonant X-ray scattering (RXS) occurs when a photon is absorbed promoting a core electron to empty states, and is subsequently re-emitted when the electron and the core hole recombine (Hannon *et al.*, 1988 ▸, 1989 ▸). The process introduces anisotropic contributions to the X-ray susceptibility tensor, whose amplitude strongly increases as the photon energy is tuned to an atomic absorption edge. The scattering amplitude also depends on the initial and final polarization of the photons. Measurements are usually performed with incident photons linearly polarized along the direction perpendicular to the scattering plane (σ polarization), whilst a polarization analyser is used to detect photons linearly polarized either along the same direction (σσ channel) or parallel to the scattering plane (σπ channel).

The first RXS measurements were performed with photons tuned to the *L* edges of holmium metal (Gibbs *et al.*, 1988 ▸), and it was soon realized that to observe the maximum magnetic effects one had to tune to energies that were associated with empty states of the spin-polarized electrons, *i.e.* *L* edges for 3*d* systems, and *M* edges for 4*f* and 5*f* systems. At the *M*_4_ edge of uranium, in the compound UAs (Isaacs *et al.*, 1989 ▸) an increase in intensity of six orders of magnitude was observed for magnetic Bragg peak when the material ordered magnetically.

For an electric dipole transition (*E*1), involving the excitation 3*d*_3/2,5/2_ → 5*f* at the *M*_4,5_ absorption edges of an actinide atom, the resonant X-ray scattering amplitude contains a scalar term, probing the electric charge, a rank-1 tensor odd in time reversal symmetry, probing the magnetic dipole moment, and a rank-2 tensor even under time reversal, probing the electric quadrupole moment,

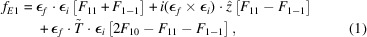

where ε_*i*,*f*_ are unit vectors giving the polarization of the incident (*i*) and scattered (*f*) photons, 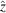 is the direction of the magnetic dipole moment, 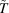 is a rank-2 tensor proportional to the electric quadrupole operators or arising from an intrinsic asymmetry of the crystal lattice, and *F*_1*q*_ (*q* = 0, ±1) are resonant energy factors (Hill & McMorrow, 1996 ▸).

If magnetic dipole moments or electric quadrupoles order, and the photon energy is large enough for diffraction to occur, the interference of the anomalous scattering amplitudes leads to the appearance of Bragg peaks at positions **Q** forbidden by the crystallographic space group. Their intensity depends to a marked extent on the energy *E* of the incident photon across the *M*_4,5_ actinide absorption edge, on the polarization of the incident and diffracted photons, and on the sample rotational orientation around the scattering vector **Q** (azimuthal angle Ψ).

In the case of electric quadrupole order, the structure factor is obtained from the electric quadrupole operators in Cartesian components, 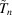 = 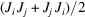 (*ijk* = *xyz*) and **J** being the angular momentum operator), as 

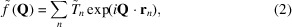

where the sum runs over all the atoms in the unit cell, at positions **r**_*n*_, and the scattering amplitude at resonance conditions is 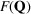 = 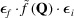 .

**Figure 5:**
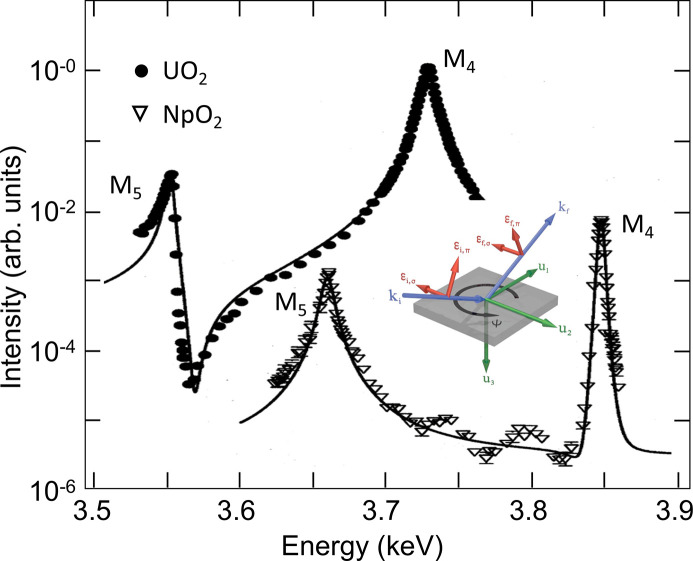
The integrated intensity of the (003) superlattice Bragg peak as a function of the photon energy around the *M*_5_ and *M*_4_ absorption edge of U and Np in UO_2_ and NpO_2_. Data were collected in the ordered phase, with the incident beam polarized perpendicularly to the diffraction plane (σ) and the scattered photon beam polarized in the diffraction plane (π). The intensity data are corrected for self-absorption. The maxima of the intensity enhancement occur at the *E*1 dipole threshold energy and are associated with 3*d*_3/2,5/2_ → 5*f* transitions. Note that the shape of the *M*_4_ resonance in NpO_2_ is a Lorentzian squared, as predicted by Nagao & Igarashi (2005 ▸). The inset on the upper side is a schematic representation of the resonant scattering process. The central inset is a sketch of the scattering geometry used to measure the integrated intensity as a function of azimuthal angle Ψ, describing the rotation of the sample about the scattering vector. The arrows ε_(*i*,*f*)(σ, π)_ correspond to the polarization direction of photons polarized perpendicularly (σ) or parallel (π) to the scattering plane. The **u**_3_ Cartesian axis is antiparallel to scattering vector **Q**.

RXS experiments provide direct evidence for the ordering of electric quadrupole moments in UO_2_ (Wilkins *et al.*, 2006 ▸), NpO_2_ (Paixão *et al.*, 2002 ▸; Caciuffo *et al.*, 2003 ▸), and in mixed U_1–*x*_Np_*x*_O_2_ solid solutions (Wilkins *et al.*, 2004 ▸). These oxides crystallize in the face-centered-cubic (f.c.c.) fluorite structure, but in the ordered phase resonant superlattice Bragg peaks appear at positions that are forbidden in the 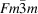 space group, such as **Q** = (00ℓ), ℓ = 2*n* + 1 (Fig. 5 ▸). The nature of the order parameter can be established by analyzing the azimuth angle dependence of their intensity in different polarization channels. As shown by equation (1), the term probing magnetic dipoles rotates the photon polarization by π/2. If measurements are performed with σ-polarized incident photons, magnetic scattering appears only in the σπ channel, whereas quadrupole scattering will contribute to both σπ and σσ channels. The intensity modulation of the *forbidden* peaks provides information on the relative orientation of the moments carried by the atoms in the base of the unit cell.

**Figure 6:**
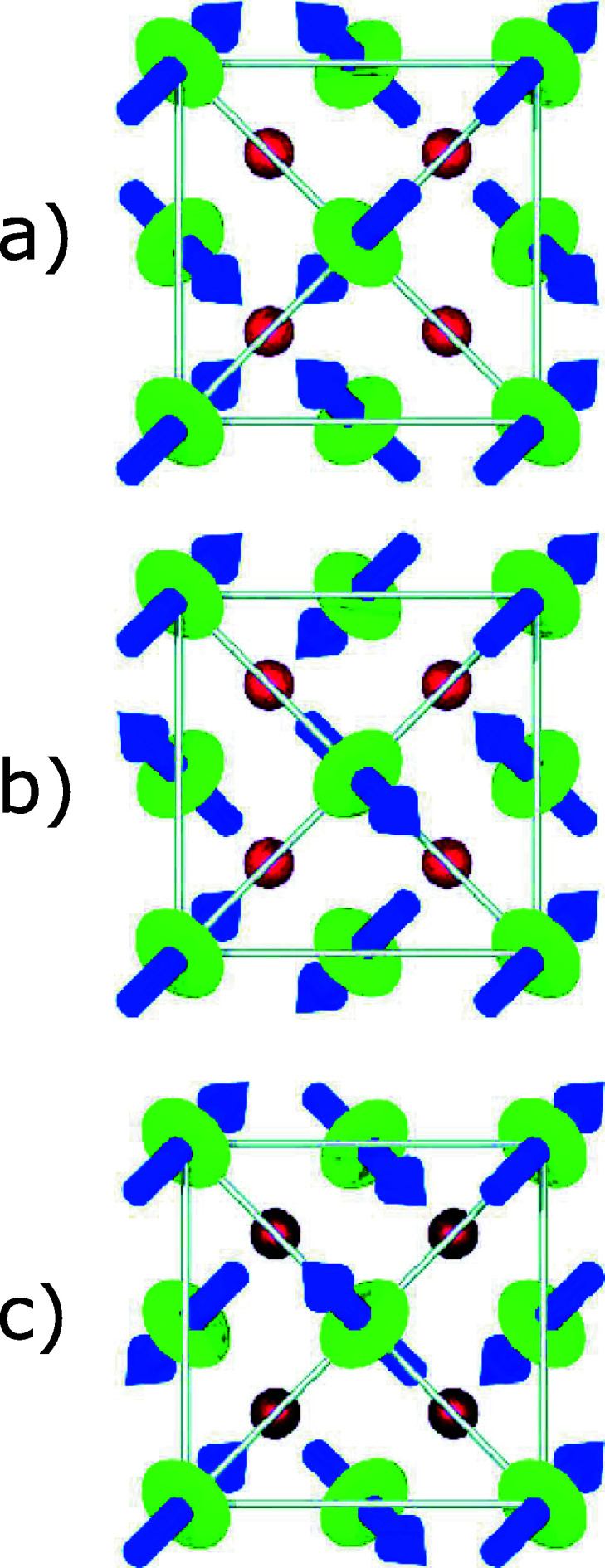
Schematic representation of the projection onto the *ab* plane of the 3-**k** magnetic and electric quadrupole ordering for the longitudinal (*a*) configuration and the two *S*-domains of the transverse configuration (*b*, *c*). The magnetic dipole moments are represented by blue arrows whereas the electric quadrupole moments are shown as green ellipsoids. The red spheres represent oxygen atoms. UO_2_ adopts the transverse structure, whereas the electric quadrupole longitudinal order is realized in NpO_2_ with zero magnetic dipole moment. Reprinted with permission from Wilkins *et al.* (2006 ▸). Copyright (2006) by the American Physical Society.

UO_2_ orders at *T*_N_ = 30.8 K. The primary order parameter is the magnetic dipole, whilst electric quadrupoles act as secondary order parameters. A 3-**k**, type-I, transverse antiferromagnetic structure with propagation vector **k** = (001) becomes stable below *T*_N_ (Burlet *et al.*, 1986 ▸; Blackburn *et al.*, 2005 ▸). The symmetry of the lattice is reduced to *Pa*3 and the uranium sublattice becomes simple cubic. Each of the four atoms in the base (*C*_2*h*_ point group) carries an electric quadrupole moment given by a linear combination of the three Γ_5_ quadrupoles transforming as *xy*, *xz*, and *yz*. The resulting order is also transverse 3-**k**. The two possible symmetry-equivalent *S* domains are shown schematically in panels (*b*) and (*c*) of Fig. 6 ▸. A visual inspection of the figure makes immediately evident that the order of the electric quadrupoles must be accompanied by an internal distortion of the oxygen sublattice as a consequence of the perturbed electrostatic interaction between the oxygen anions and the asymmetric 5*f* electronic cloud around the uranium ions. This distortion of the oxygen atoms in UO_2_ was first reported (Faber *et al.*, 1975 ▸) in 1975 using neutron scattering, but the reason for the distortion was not understood at that time. Neutrons cannot observe the electric quadrupoles.

In NpO_2_, a second order phase transition is observed at *T*_0_ = 25 K. In this case, the crystallographic structure is preserved and neither external nor internal distortions are observed (Caciuffo *et al.*, 1987 ▸). The Ψ dependence of the (001) and (003) Bragg peaks intensity has been measured with the sample kept at *T* = 10 K at the maximum of the *M*_4_ absorption edge (*E* = 3.846 keV) (Paixão *et al.*, 2002 ▸). Data collected in the σπ and σσ channels have been used to obtain the Stokes parameters 

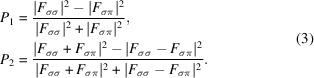

**Figure 7:**
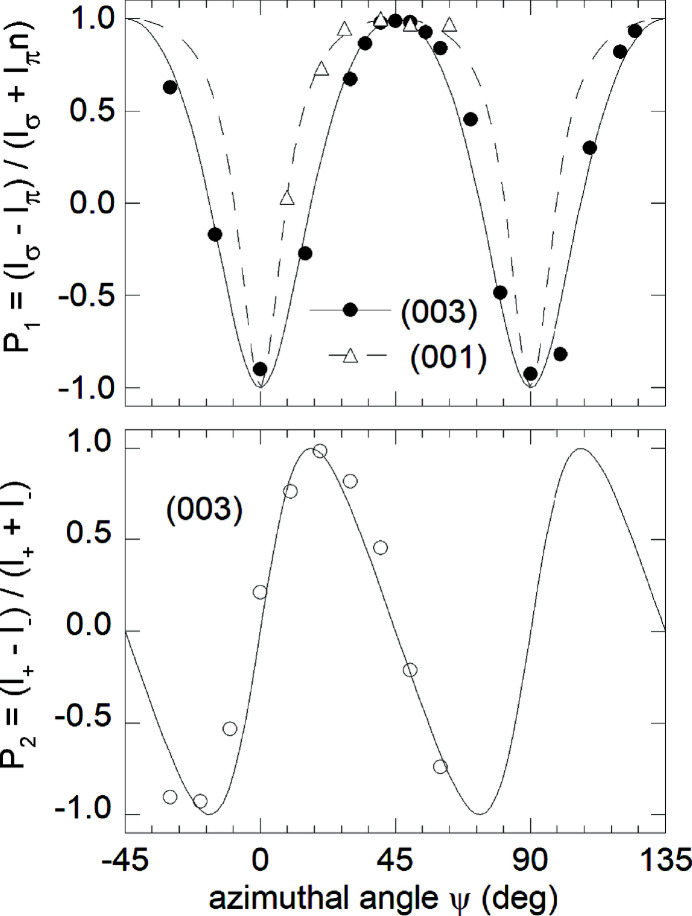
Azimuthal angle dependence of the Stokes parameters *P*_1_ and *P*_2_ for the (001) and (003) superlattice reflections measured in NpO_2_ at 10 K with *E* = 3.846 keV. The origin of the azimuthal angle Ψ corresponds to the *a*-axis lying in the scattering plane. Lines are calculations based on a longitudinal, 3-**k** order of Γ_5_ electric quadrupoles with zero ordered magnetic dipole moments. No fitting parameter are involved. Republished with permission of IOP Publishing from Caciuffo *et al.* (2003 ▸); permission conveyed through Copyright Clearance Center, Inc.

Fig. 7 ▸ shows the experimental results. The lines in the figure correspond to calculations assuming the longitudinal 3-**k** order of Γ_5_ electric quadrupoles shown in panel (*a*) of Fig. 6 ▸, assuming a zero ordered magnetic dipole moments (Caciuffo *et al.*, 2003 ▸).

For a **Q** = (0 0 ℓ) reflection, choosing Ψ = 0 where the [100] vector is in the scattering plane with a component parallel to the incident photon beam, the azimuthal and polarization dependence of the RXS amplitude for the considered structure are 

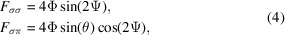

where Φ is the quadrupole order parameter. Therefore, 

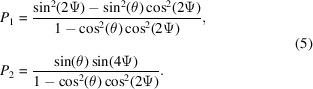

The agreement between experimental and calculated data in Fig. 7 ▸ is excellent, considering that no fitting parameters are used. As a consequence of the quadrupole order, the point symmetry at the Np site is reduced to *D*_3*d*_ and the space group is lowered to 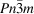 . However, with Np ions on 4*b* and O ions on 2*a* and 6*d* Wyckoff positions, the crystallographic extinction rules remain the same as those of the 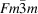 space group. Also for NpO_2_ quadrupoles are not the primary order parameter. Indeed, by probing the dynamics of the ordered state by inelastic neutron scattering it appears that the driving order parameter is provided by the rank-5 magnetic tri­akontadipoles (Santini *et al.*, 2006 ▸; Magnani *et al.*, 2008 ▸).

Searches for quadrupolar order by RXS experiments have been performed also on URu_2_Si_2_. This intermetallic compound has been widely investigated (Mydosh *et al.*, 2020 ▸) in an attempt to explain the nature of its phase transition at *T*_0_ = 17.5 K. The puzzle arises from the difficulty in reconciling the tiny value of the ordered magnetic moment (μ_0_ ≃ 0.03μ_B_ along the *c*-axis of the tetragonal unit cell) with the large macroscopic anomalies observed at *T*_0_ (Broholm *et al.*, 1991 ▸; Mason *et al.*, 1990 ▸; Walker *et al.*, 1993 ▸). For instance, if the order parameter were the magnetic dipole moment, the anomaly in the specific heat should be about 30 times smaller. This indicates that macroscopic anomalies are not associated with μ_0_, but rather with a *hidden* order parameter not directly coupled to scattering probes. Among a number of theoretical models (Oppeneer *et al.*, 2011 ▸; Mydosh *et al.*, 2020 ▸), a staggered ordering of electric quadrupoles has been suggested to occur in URu_2_Si_2_ (Santini & Amoretti, 1994 ▸; Santini *et al.*, 2000 ▸).

**Figure 8:**
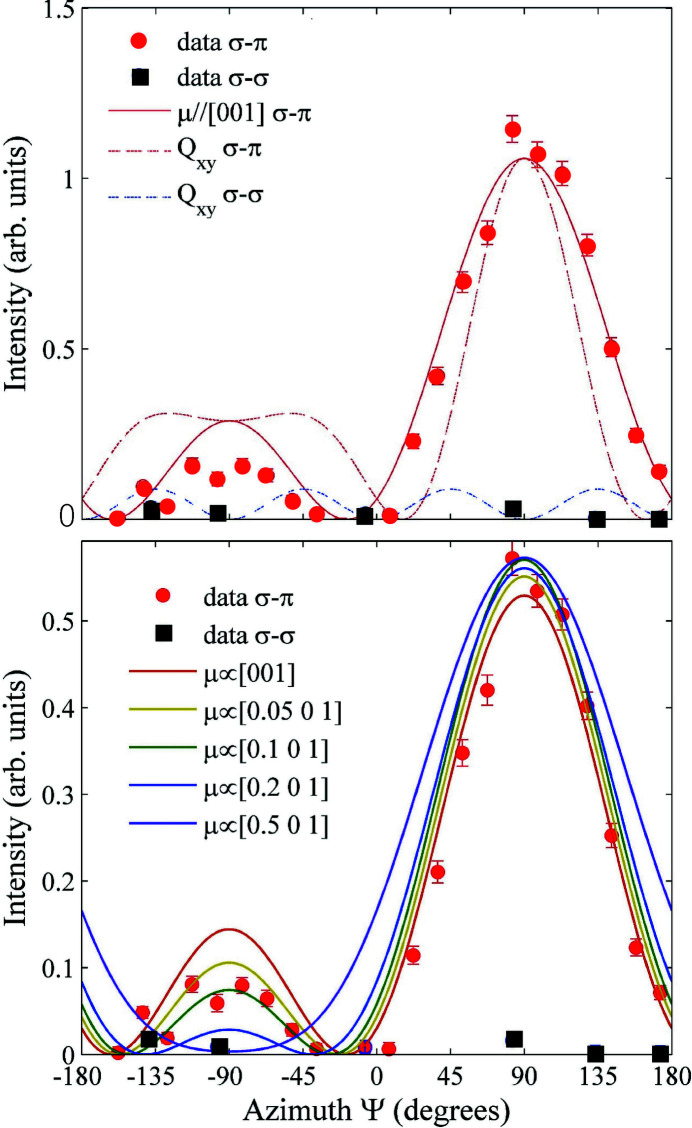
Top panel: azimuthal dependence of the (201) Bragg reflection in URu_2_Si_2_. Solid (red) circles: σπ channel; black squares: σσ channel. The full (red) line is the theoretical intensity variation for magnetic dipoles ordered along [001]. The dashed red (dashed blue) line is the σπ (σσ) Ψ dependence of the intensity expected for *xy* quadrupole order. Bottom panel: experimental data compared with theoretical predictions for a dipole magnetic moment with an increasing component in the *ab* crystallographic plane. Reprinted with permission from Walker *et al.* (2011 ▸). Copyright (2011) by the American Physical Society.

RXS experiments at the uranium *M*_4_ absorption edge have not confirmed this hypothesis (Walker *et al.*, 2011 ▸). Data have been collected on a high-quality single crystal of URu_2_Si_2_, cut with a [101] direction specular, spanning an extended region of the reciprocal space plane [*H*0*L*]. The results exclude electric quadrupoles of any symmetry as a hidden-order parameter with a propagation vector in the explored region. Indeed, as shown in the top panel of Fig. 8 ▸, forbidden Bragg peaks emerging in the ordered state have non-zero intensity only in the σπ polarization channel, with an azimuthal angle dependence corresponding to an ordered magnetic dipole moment along the crystallographic *c*-axis. The bottom panel of Fig. 8 ▸ shows that the Ψ dependence of the superlattice peak intensity is very sensitive to the inclusion of a component of the ordered magnetic moment in the *a* *b* plane, whose estimated upper limit is ∼0.003μ_B_. This experimental result essentially eliminates the idea of hastatic order proposed by Chandra *et al.* (2015 ▸).

The capability of the RXS technique to detect higher-order electric multipoles has, of course, been of great interest and further examples are given by Santini *et al.* (2009 ▸). Important early work was done on UPd_3_ (McMorrow *et al.*, 2001 ▸; Walker *et al.*, 2006 ▸), and certainly more materials will be discovered with such quadrupole ordering. An example of Templeton scattering has recently been discovered in a rather complicated actinide system U_2_N_3_ (Lawrence Bright *et al.*, 2019 ▸), and, since it was observed at the *M*_4_ edge of uranium, this suggests a participation of the 5*f* states in the bonding of this material, although the precise form has not yet been determined.

The enormous enhancement of the magnetic signal at the actinide *M* edges has also been of interest in many actinide materials to learn more about the magnetic structure itself. Early work, for example, determined in greater detail the nature of the multi-*k* structures found in materials with transuranium ions (Langridge *et al.*, 1994*a* ▸,*b* ▸; Normile *et al.*, 2002*a* ▸,*b* ▸; Lidström *et al.*, 2000 ▸), as well as those with uranium (Longfield *et al.*, 2002 ▸; Bernhoeft *et al.*, 2004*a* ▸), and thin films (Bernhoeft *et al.*, 1998 ▸; Bao *et al.*, 2013 ▸). The large intensity also allowed experiments to probe surface magnetism in UO_2_ showing new examples of surface ordering not previously measured (Watson *et al.*, 1996 ▸, 2000 ▸; Langridge *et al.*, 2014 ▸). Similarly, the large intensity led to the discovery of an interesting effect that appears to occur when a magnetic material disorders. No satisfactory explanation of this effect has yet been proposed (Bernhoeft *et al.*, 2004*b* ▸). After a considerable effort this small effect was also seen with neutrons in the antiferromagnet MnF_2_, showing that it is not limited to actinides, and is not related to a surface effect (Prokeš *et al.*, 2009 ▸).

## 4. X-ray absorption spectroscopy

X-ray absorption spectroscopy (XAS) of actinides at synchrotron radiation sources started in the late 1980s, exploring both the *L*_2,3_ (2*p* → 6*d*) (Kalkowski *et al.*, 1987*a* ▸; Bertram *et al.*, 1989 ▸) and the *M*_3,4,5_, *N*_4,5_, and *O*_4,5_ (*n* *d* → 5*f*; *n* = 3,4,5) (Kalkowski *et al.*, 1987*b* ▸) absorption edges. The potential of XAS for elucidating the electronic structure of actinides was immediately recognized and a number of studies on actinide speciation in compounds and minerals followed (Silva & Nitsche, 1995 ▸; Conradson *et al.*, 1998 ▸; Denecke, 2006 ▸). This body of experimental work stimulated the construction of dedicated beamlines, such as CAT-ACT at ANKA (Dardenne *et al.*, 2009 ▸; Zimina *et al.*, 2017 ▸) and ROBL-II at ESRF (Scheinost *et al.*, 2021 ▸). Today, XAS experiments at state-of-the-art spectrometers have been extended to the heaviest actinides available in macroscopic quantities, as recently demonstrated by a study of a coordination complex of ^254^Es that used less than 200 ng of this highly radioactive isotope (half-life of ∼275.7 days) (Carter *et al.*, 2021 ▸).

Besides the scientific importance, actinide speciation studies are key to understanding how these elements can contaminate the environment and how best they can be removed. The clean up of the Rocky Flats Nuclear Weapons Plant, near Denver, USA, is exemplary of the practical importance of these studies (Clark *et al.*, 2006 ▸).

From the point of view of physics, XAS experiments at the *M*_4,5_ and *N*_4,5_ absorption edges of actinides have been important to probe the relativistic nature of the 5*f* electrons, thanks to the application of simple spin–orbit sum rules relating the branching ratio of the core-valence transitions to the expectation value of the angular part of the 5*f* spin–orbit interaction per hole (Thole & van der Laan, 1988 ▸; van der Laan *et al.*, 2004 ▸; Shim *et al.*, 2009 ▸; Caciuffo *et al.*, 2010*a* ▸). The information obtained is analog to that provided by electron energy-loss spectroscopy experiments (EELS) (Moore & van der Laan, 2009 ▸), a technique that proved to be very useful in elucidating the change between α and δ plutonium (Moore *et al.*, 2006 ▸), the magnetic stabilization in curium metal (Moore *et al.*, 2007 ▸), or to study rare metals only available in small quantities (Dieste *et al.*, 2019 ▸).

## 5. X-ray magnetic circular dichroism

XMCD is associated with time-reversal symmetry breaking by a magnetic field and involves electric dipole or electric quadrupole transitions promoting an electron in a spin–orbit split core state to an empty valence state of the absorbing atom. The technique provides an element- and shell-specific probe for studying the electronic structure of a wide range of materials (van der Laan, 2013 ▸). In actinides materials, XMCD spectra are conveniently measured at the *M*_4,5_ (3*d*_3/2,5/2_ → 5*f*) absorption edges that directly interrogate the 5*f* shell. The XMCD signal is obtained as the difference 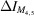 = 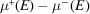 of the absorption spectra of circularly polarized photons with helicity parallel [μ^+^(*E*)] and antiparallel [μ^−^(*E*)] with respect to a magnetic field applied along the incident beam direction. The spectra must be corrected for self-absorption effects and for the incomplete polarization of the incident beam emerging from the crystal monochromator. Standard protocols have been developed for performing such corrections (Goulon *et al.*, 1982 ▸; Tröger *et al.*, 1992 ▸; Pfalzer *et al.*, 1999 ▸).

The power of the technique comes from the simplicity of the sum rules relating orbital and spin moments of the absorbing atoms to linear combinations of the dichroic signal integrated over the pair of spin–orbit-split excitations, 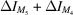 , normalized to the isotropic X-ray absorption spectrum (Thole *et al.*, 1992 ▸; Carra *et al.*, 1993 ▸; van der Laan & Thole, 1996 ▸; van der Laan *et al.*, 2004 ▸), 

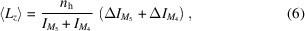




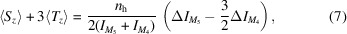

where 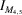 is the integrated intensity of the isotropic X-ray absorption spectrum, and *n*_h_ is the number of holes in the 5*f* shell. The magnetic dipole term *T*_*z*_ is the component along the quantization axis of an operator associated with charge and magnetic anisotropy (Collins *et al.*, 1995 ▸) and correlating spin **s**_*i*_ and position **r**_*i*_ of individual electrons, **T** = 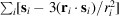 . The orbital and spin components of the total magnetic moment of the 5*f*-shell, μ = −(〈*L*_*z*_〉 + 2〈*S*_*z*_〉), can then be obtained from XMCD spectra, together with an estimate of 〈*T*_*z*_〉, if the value of the total moment μ and the occupation number of the 5*f* shell are known.

Furthermore, the expectation value of the angular part of the valence states spin–orbit operator, 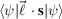 , can be obtained from the XAS branching ratio, *B* = 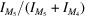 , as (Thole & van der Laan, 1988 ▸) 

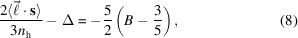

where Δ is a quantity dependent on the electronic configuration (van der Laan *et al.*, 2004 ▸) (for Np^3+^, for instance, Δ = −0.005).

The XAS and XMCD experiments reviewed here were carried out at the ID12 beamline of the European Synchrotron Radiation Facility (ESRF) using samples encapsulated in an Al holder with Kapton windows of 60 µm thickness in total. The available cryomagnet affords magnetic fields up to 17 T and a base temperature of about 2 K. The minimum mass of the sample depends on the magnetic susceptibility of the system. For elemental curium, data have been collected on a 0.55 mg sample of ^248^Cm (Lander *et al.*, 2019 ▸), whereas a sample mass of 16 µg was sufficient for Am in a sample of AmFe_2_ (Magnani *et al.*, 2015 ▸).

**Figure 9:**
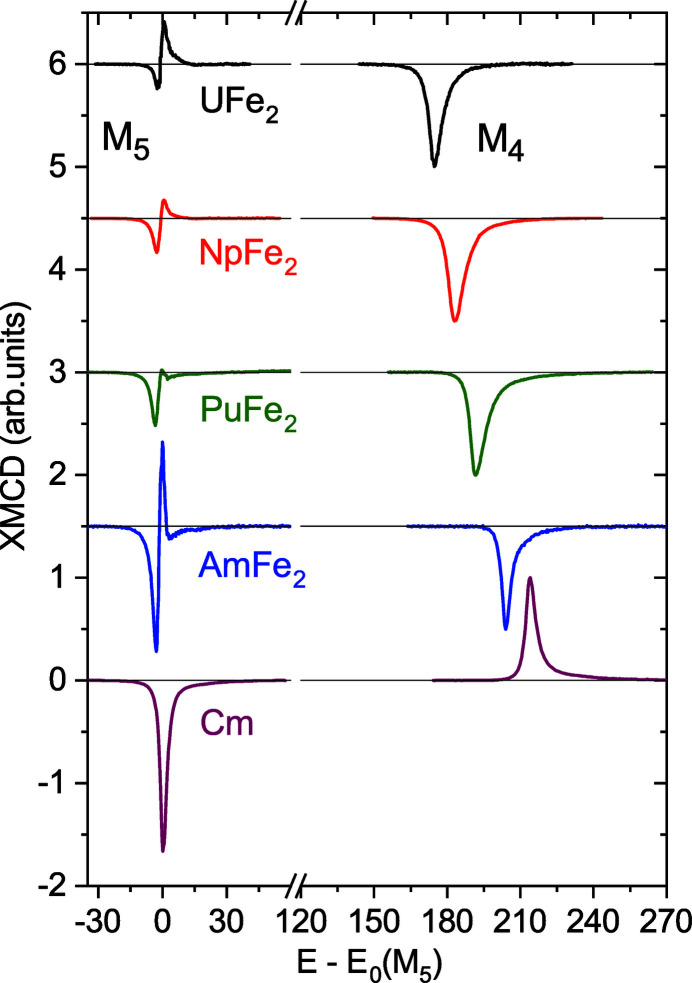
XMCD spectra as a function of photon energy through the An *M*_4,5_ edges in AnFe_2_ (An = U, Np, Pu, Am) and elemental Cm. The spectra have been corrected for self-absorption effects and incomplete circular polarization of the incident beam. Adapted from Lander *et al.* (2019 ▸).

Fig. 9 ▸ shows an overview of the XMCD signals measured at the *M*_5_ and *M*_4_ edges in ferromagnetic AnFe_2_ (An = U, Np, Pu, Am) compounds (Wilhelm *et al.*, 2013 ▸; Magnani *et al.*, 2015 ▸). These spectra are compared with the XMCD signal measured for elemental curium (Lander *et al.*, 2019 ▸). For convenience, the photon energy is set to zero at the *M*_5_ edge and the amplitude at the *M*_4_ edge is normalized to unity. It must be noticed that the spectral intensity at *M*_4_ actually becomes smaller with increasing atomic number from uranium to americium, as the number of holes in the *j* = 5/2 subshell decreases. Moreover, in absolute units the XMCD signal for AmFe_2_ is smaller than for NpFe_2_, reflecting the difference between the magnetic moments in the two compounds, whereas the narrower linewidth of the *M*_4_ line in AmFe_2_ indicates the presence of localized 5*f* states, as observed in PuSb (Janoschek *et al.*, 2015 ▸).

In the case of curium, a visual inspection of the XMCD spectra is sufficient to realize that the integrated intensity of the features at *M*_5_ and *M*_4_ are not equal. From the first sum rule, this means that the orbital moment is not zero. The value provided by the experiment at 70 K is μ_*L*_ = −〈*L*_*z*_〉 = 0.10 (1)μ_B_, with a ratio μ_*L*_/μ_*S*_ = +0.06 close to the calculated value of +0.052 for the *f* ^7^ configuration in intermediate coupling (van der Laan & Thole, 1996 ▸). The fact that μ_*L*_ and μ_*S*_ are parallel in Cm metal is the reason why the sign of the *M*_4_ line suddenly changes in comparison with the earlier elements (Fig. 9 ▸).

**Figure 10:**
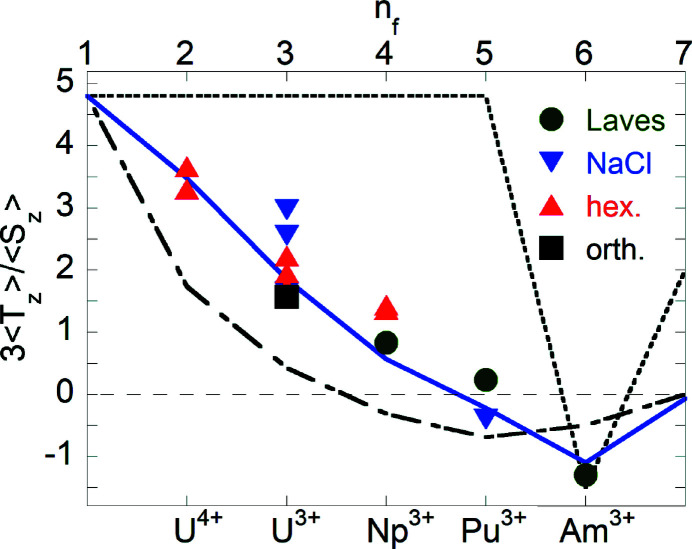
The ratio 3〈*T*〉_*z*_/〈*S*〉_*z*_ between the expectation values of the magnetic dipole and the spin operators along the quantization direction as a function of the 5*f* occupation number *n*_*f*_. Symbols are experimental data obtained from an analysis of XMCD spectra at the *M*_4,5_ actinide edges for compounds with different physical properties and crystallographic structure (circles for C-15 cubic Laves phases, squares for orthorhombic structures, down triangles for NaCl-type structure, and up triangles for hexagonal lattice groups). Theoretical estimates are shown for intermediate coupling (IC) (solid lines), Russell–Saunders (LS) (dashed line), and *j*–*j* (dotted line) coupling approximations. Reprinted with permission from Magnani *et al.* (2015 ▸). Copyright (2015) by the American Physical Society.

Equation (7) shows that the spin component of the magnetic moment can only be obtained if the value of 〈*T*_*z*_〉 is known. This quantity cannot be measured directly. However, it can be immediately obtained from an analysis of the XMCD spectra if an independent measurement of the total magnetic moment μ = μ_*S*_ + μ_*L*_ is available, for instance from neutron diffraction experiments or, in the case of Np compounds, from ^237^Np Mößbauer spectroscopy. Fig. 10 ▸ shows the 3〈*T*_*z*_〉/〈*S*_*z*_〉 ratio obtained for several compounds for which such information was available. By changing the 5*f* occupation number *n*_*f*_, the ratio 3〈*T*_*z*_〉/〈*S*_*z*_〉 varies as predicted by atomic calculations in the intermediate coupling (IC) approximation. The correlation is very convincing and suggests that 〈*T*_*z*_〉 can be reliably estimated by IC calculations when the total magnetic moment μ is not known.

**Figure 11:**
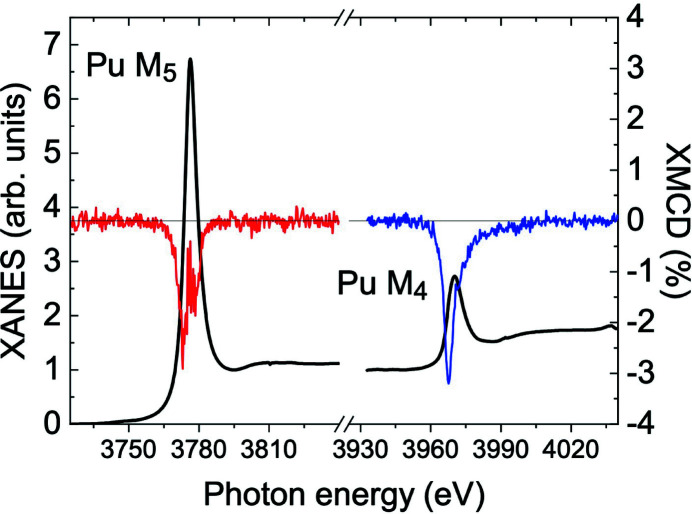
XANES (solid black lines) and XMCD spectra as a function of photon energy through the Pu *M*_5_ (red line) and *M*_4_ (blue line) edges in PuCoGa_5_. Reprinted with permission from Magnani *et al.* (2017 ▸). Copyright (2017) by the American Physical Society.

This is, for instance, the case when the absorbing atom is located inside a vortex in a type-II superconducting compound. As an example, Fig. 11 ▸ shows XANES and XMCD spectra measured in PuCoGa_5_ (Magnani *et al.*, 2017 ▸), a tetragonal heavy fermion superconductor with a critical temperature *T*_c_ = 18.5 K (Sarrao *et al.*, 2002 ▸). The origin of superconductivity in this compound remains puzzling, despite years of intensive investigations (Jutier *et al.*, 2008 ▸; Das *et al.*, 2015 ▸; Ramshaw *et al.*, 2015 ▸). The Pu ground state is non-magnetic (Hiess *et al.*, 2008 ▸), and the superconducting order parameter has *d*-wave symmetry (Daghero *et al.*, 2012 ▸).

XMCD spectra have been measured above and below *T*_c_ on a 2 mg single-crystal sample of ^242^PuCoGa_5_ (99.99 wt% ^242^Pu) (Magnani *et al.*, 2017 ▸). The *c*-axis of the crystal was oriented along the incident photon beam, and therefore parallel to the applied magnetic field. The critical field *B*_c2_(*T* = 0) along the *c*-axis is 63 T, so that a vortex phase is present in an applied field smaller than 15 T. The 5*f* shell of the Pu atoms inside the vortex cores is polarized by the external field and the XMCD response is different from zero. Applying the sum rules, the moments and their ratio can be extracted from the experimental data. The values obtained are in excellent agreement with dynamical mean field theories (DMFT) predictions of a non-magnetic ground state for Pu in PuCoGa_5_ [see Table I of Magnani *et al.* (2017 ▸)]. This surprising result is a consequence of two effects: intermediate valence driven by 5*f*–6*d* Coulomb interaction mixes a magnetic 5*f* ^5^ sextet with a non-magnetic 5*f* ^6^ singlet, reducing the magnetic moment; a complete quenching is then produced by a Kondo-like screening promoted by the hybridization between 5*f* and conduction electron states (Pezzoli *et al.*, 2011 ▸; Shick *et al.*, 2013 ▸).

## 6. Non-resonant inelastic X-ray scattering

Non-resonant inelastic X-ray scattering (NIXS) at the *O*_4,5_ (5*d*_3/2,5/2_ → 5*f*) absorption edges is a bulk-sensitive technique exploiting multipole transitions from core 5*d* to valence 5*f* states. For small values of the scattering vector, *Q*, the NIXS spectra are dominated by the dipole-allowed transitions encapsulated within the giant Fano resonance. For high *Q* values, the intensity of the dipole transitions becomes negligible and the spectral response is dominated by dipole-forbidden transitions appearing as well resolved multiplet structure in the pre-edge region (Macrander *et al.*, 1996 ▸; Gurtubay *et al.*, 2005 ▸; Larson *et al.*, 2007 ▸; Haverkort *et al.*, 2007 ▸; van Veenendaal & Haverkort, 2008 ▸; van der Laan, 2012*a* ▸). These features provide information on states with symmetries different than those interrogated by electric dipole transitions in XAS, and are sensitive to atomic environment, valence, and hybridization effects (Gordon *et al.*, 2008 ▸).

Compared with other X-ray spectroscopy techniques, NIXS is intrinsically intensity-limited. On the other hand, the weakness of the involved photon–matter interaction process and the absence of an intermediate state make it easy to model the experimental data in a quantitative way. Another advantage of NIXS is that shallow energy edges are probed by hard X-rays. Bulk-sensitive information is, therefore, obtained. Moreover, the high penetration depth of hard X-rays (∼5 µm in UO_2_ for 10 keV incident photons) makes feasible the study of encapsulated samples, which is a prerequisite in the case of transuranium materials.

Far away from resonance conditions, the radiation–matter interaction is dominated by a term proportional to the square of the vector potential **A**. Within the first Born approximation, the NIXS double differential cross section is obtained by expanding the transition operator 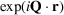 in terms of spherical harmonics, and is given by (Schülke, 2007 ▸; Haverkort *et al.*, 2007 ▸; Gordon *et al.*, 2008 ▸) 

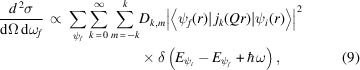

where **Q** = **k**_*i*_ − **k**_*f*_ is the scattering vector, **k**_*i*,*f*_ are the wavevectors of the incident and scattered radiation, *D*_*k*,*m*_ is a term describing the angular dependence of the signal, and *j*_*k*_(*Qr*) are spherical Bessel functions of rank *k*. The multipole moments *k* for the ℓ → ℓ′ shell transition are limited by the triangular condition, |ℓ − ℓ′| ≤ *k* ≤ ℓ + ℓ′, and the parity rule, ℓ + ℓ′ + *k* = even. Thus for *d* → *f* transitions, only *k* = 1 (dipole), *k* = 3 (octupole), and *k* = 5 (triakontadipole) transitions are allowed. The interrogated final-state *J*′ and *L*′ are restricted by the conditions |*J* − *k*| ≤ *J*′ ≤ *J* + *k* and |*L* − *k*| ≤ *L*′ + Δ*S* ≤ *L* + *k*. Here, Δ*S* = 0 in *LS* coupling and Δ*S* = ±1 in intermediate coupling. Lower energy states with high *L*′ and *J*′ values can therefore be reached by higher multipole transitions.

The potential of NIXS for characterizing the dynamical electron-density response in actinide materials has been demonstrated in a few studies of oxides and intermetallic compounds at the *O* absorption edges (Caciuffo *et al.*, 2010*b* ▸; Bradley *et al.*, 2010 ▸; Sen Gupta *et al.*, 2011 ▸; Sundermann *et al.*, 2016 ▸, 2018 ▸, 2020 ▸). Attempts to carry out NIXS experiments at the *N* edges of actinides have been frustrated by the weakness of the signal.

**Figure 12:**
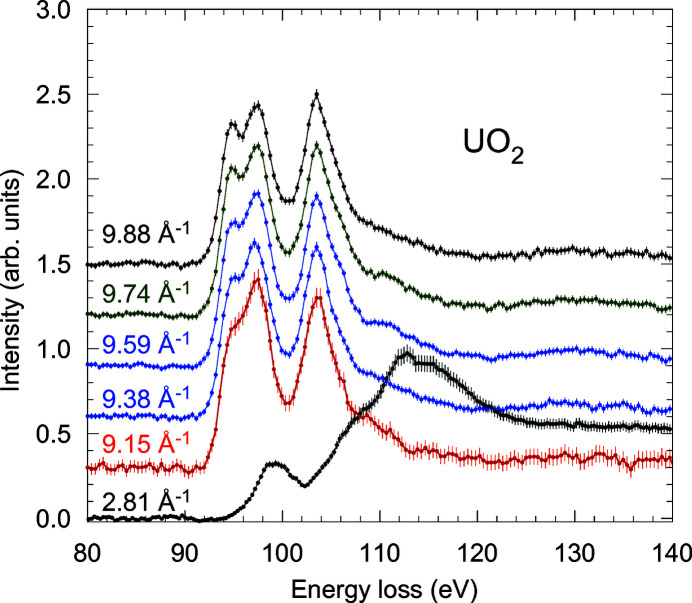
NIXS spectra measured for UO_2_ at the uranium *O*_4,5_ edges with a fixed final energy of 9.689 keV, at different values of the scattering vector *Q*. Data at different *Q* values are normalized to the peak intensity of the feature centered at about 104 eV. Reprinted with permission from Caciuffo *et al.* (2010*b* ▸). Copyright (2010) by the American Physical Society.

Fig. 12 ▸ shows, as an example, the NIXS room-temperature spectra obtained for UO_2_ in an energy loss (ℏω = *E*_*i*_ − *E*_*f*_) range encompassing the uranium *O*_4,5_ absorption edges (Caciuffo *et al.*, 2010*b* ▸). Data were collected at the ID16 inverse-geometry, multiple-analyzer-crystal spectrometer (Verbeni *et al.*, 2009 ▸) of ESRF, with a resolution of ∼1.3 eV at a final photon energy *E*_*f*_ of 9.689 keV. The sample was a UO_2_ single crystal with the exposed external surface perpendicular to [111].

At *Q* = 2.81 Å^−1^, the response is dominated by the dipole transition and shows the broad, asymmetric Fano profile due to resonant decay into continuum states, as observed by XAS measurements (Kalkowski *et al.*, 1987*b* ▸). Above *Q* ≃ 9 Å^−1^, the higher multipole transitions appear at lower energies, with three resolved features at 94.9, 97.3, and 103.6 eV at *Q* = 9.88 Å^−1^, providing a fingerprint for the uranium ground state (the radial and angular dependence of the NIXS cross section for the *O*-edge of a U^4+^ 5*f* ^2^ configuration is discussed by Sundermann *et al.* (2016 ▸) and Caciuffo *et al.* (2010*b* ▸).

An excellent agreement was found between the experimental data shown in Fig. 12 ▸ and many-electron atomic spectral calculations in intermediate coupling that allows one to identify the origin of the observed multipole transitions (Caciuffo *et al.*, 2010*b* ▸, 2012*a* ▸). Similar experiments were also extended to transuranium materials, investigating 5*f* ^3^, 5*f* ^4^, and 5*f* ^5^ configurations in NpO_2_, PuO_2_, and β-Pu_2_O_3_ (Sundermann *et al.*, 2020 ▸).

**Figure 13:**
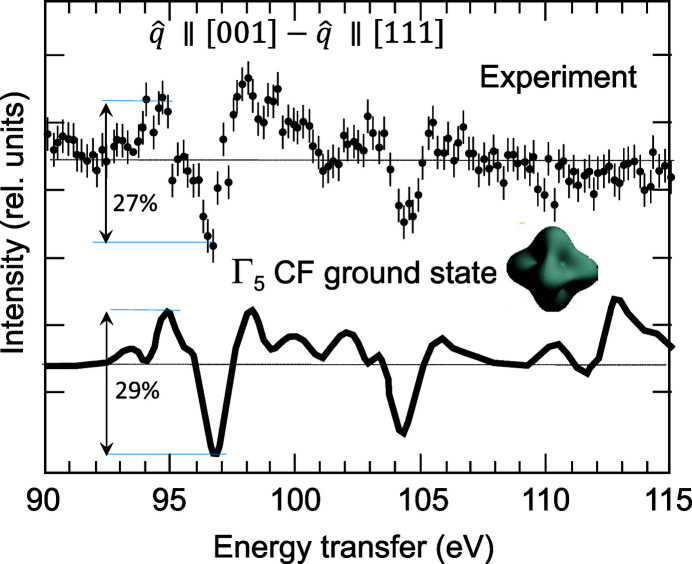
Difference between the experimental NIXS spectra measured at the uranium *O*_4,5_ edges in single-crystal UO_2_ with momentum transfer direction 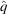 = 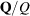 along [001] and [111]. Calculations assuming a Γ_5_ triplet crystal field ground state and a crystal field potential strength compatible with inelastic neutron scattering experiments are shown by the solid line. Reprinted with permission from Sundermann *et al.* (2018 ▸). Copyright (2018) by the American Physical Society.

Contrary to the dipole transition, the multipole transitions in the NIXS process branch to representations with distinct angular dependence. This fact introduces an anisotropy of the NIXS signal, with measurable differences of the spectral intensity along different directions of the momentum transfer. Such a directional dichroism can be exploited for gaining information about symmetry and strength of the crystal-field acting on the scattering atoms (Willers *et al.*, 2012 ▸). The potential of the method has been demonstrated for the tetragonal URu_2_Si_2_ intermetallic (Sundermann *et al.*, 2016 ▸) and for cubic UO_2_ (Sundermann *et al.*, 2018 ▸). Fig. 13 ▸ shows the comparison between experimental data and simulations in the case of UO_2_.

The sum rule for the branching ratio of the electric multipole transitions from a core hole to a spin–orbit split valence state probed by NIXS has been derived by van der Laan (2012*b* ▸). It shows that the effect of the valence spin–orbit interaction on the branching ratio depends on *k*, the rank of the transition. These effects are very large at the end of the transition series, as in the case of the Er 4*f* ^11^ *M*_4,5_ spectra, but are observable also for light actinides, as for the U 5*f* ^2^ *O*_4,5_ transition, providing additional information about the electronic structure of the investigated material (van der Laan, 2012*b* ▸).

## 7. Resonant inelastic X-ray scattering

**Figure 14:**
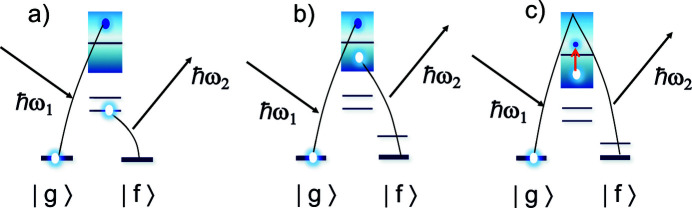
Schematic representation of direct (*a*, *b*) and indirect (*c*) RIXS processes.

Resonant inelastic X-ray scattering (RIXS) experiments consist of measuring the energy dependence of the emission line after creating a core hole by photons tuned around an atomic absorption edge (Kotani & Shin, 2001 ▸; Ghiringhelli *et al.*, 2004 ▸; Braicovich *et al.*, 2010 ▸; Rueff & Shukla, 2010 ▸; Ament *et al.*, 2011 ▸). As shown schematically in Fig. 14 ▸, RIXS is a two-step process involving first the absorption of a photon and the promotion of a core electron into an empty valence state, followed by the emission of a photon with smaller energy and the filling of the core hole by an electron sitting either in a core level [*direct core-to-core* RIXS, Fig. 14 ▸(*a*)] or in the occupied valence band [*direct valence-to-core* RIXS, Fig. 14 ▸(*b*)]. The process therefore probes the convolution between empty (absorption step) and occupied (emission step) density of states and leaves an electron–hole excitation in the final state. A different RIXS process is also possible, upon which the electron ejected from the core level and promoted above the Fermi energy refills the core-hole, after the creation of a hole–electron excitation in the valence band [*indirect* RIXS, Fig. 14 ▸(*c*)].

In a RIXS experiment, the scattering cross-section is measured by scanning the incident and emitted photon energy plane in a region around the selected absorption edge and emission transition. In the case of actinides, the combinations used in the majority of the experiments performed so far are the *L*_3_ − *L*α_1_(*L*β_5_) and the *M*_4,5_ − *M*(α,β) ones. The former, spanning the incident energy range ∼17–19 keV and an energy transfer range ∼3–4 keV, implies an absorption transition 2*p* ^6^6*d* ^*n*^ → 2*p* ^5^6*d* ^*n*+1^ followed by the emission transition 3*d* ^10^(5*d* ^10^)6*d* ^*n*+1^ → 2*p* ^6^3*d* ^9^(5*d* ^9^)6*d* ^*n*+1^. The latter, in the incident energy range ∼3.5–4.3 keV and energy transfer range ∼0.3–0.4 keV, probes the 3*d* ^10^5*f* ^*n*^ → 3*d* ^9^5*f* ^*n*+1^ absorption followed by a 4*f* ^14^5*f* ^*n*+1^ → 3*d* ^10^4*f* ^13^5*f* ^*n*+1^ emission.

Incident energy scans at the maximum of the emission line are called *high-energy resolution fluorescence detected X-ray absorption near-edge structure* (HERFD-XANES) spectra. On the other hand, measurements at fixed incident energy and varying final energy are referred to as *resonant X-ray emission spectroscopy* (RXES) (Rueff & Shukla, 2010 ▸; Vitova *et al.*, 2010 ▸; Kvashnina *et al.*, 2014 ▸). Compared with conventional XANES, HERFD-XANES offers the advantage of an increased resolution in the absorption process, since the final transitions are from intermediate states with larger intrinsic core-hole interaction lifetime. At the U *L* edge, for instance, the energy resolution is improved from ∼8 to ∼4 eV, whilst energy resolutions of 0.4–0.3 eV can be obtained at the U *M*_4,5_ edges (Kvashnina *et al.*, 2014 ▸).

At the *L*_3_ edge and the *L*α_1_ emission line, HERFD-XANES and RXES have been used to study the multiconfigurational nature of 5*f* orbitals in several uranium and plutonium intermetallics and oxides, demonstrating that the edge position can be related to the density of states at the Fermi level and that the contributions from different 5*f* electron configurations can be estimated from an analysis of the spectral features (Booth *et al.*, 2012 ▸, 2016 ▸; Tobin *et al.*, 2015 ▸).

As both incident and scattered photon energies are high in a RXES measurement at the *L*_3_ edge of actinides, this technique has been applied to study the evolution of the 5*f* states upon compression. Being sensitive to final-state energy distributions in the presence of a core-hole, RXES is a good probe of mixed-valency (Rueff & Shukla, 2010 ▸). Experiments have been performed on localized magnetic (UPd_3_) and heavy fermion (UPd_2_Al_3_) compounds, showing for the first a stable U^4+^ valence state, for the second a progressive transformation upon compression from a mixed valent U^4−δ^ state to a U^4+δ^ configuration (Rueff *et al.*, 2007 ▸). This kind of measurement in diamond anvil pressure cells requires a very small amount of material, usually 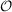 (100 µg). This is particularly welcome when working on transuranium materials because it eases the management of the safety risk. For instance, an experiment at the *L*_3_ edge of elemental americium (18.518 keV) was performed using a wide-aperture membrane-type diamond-anvil cell with a ∼5 µm-thick foil of ^243^Am metal loaded into the hole (130 µm diameter) of an inconel gasket (Heathman *et al.*, 2010 ▸). The energy dependence of the *L*α_1_ emission line (14.625 keV) was measured for pressure ranging from 4 GPa (double-hexagonal closed-packed Am phase I) to 23 GPa, into the orthorhombic Am IV phase (see Fig. 2 ▸). 5*f* electrons are supposed to be localized in Am-I and itinerant in Am-IV (Heathman *et al.*, 2000 ▸; Griveau *et al.*, 2005 ▸). Many-body electronic structure calculations, based on the dynamical mean field theory approximation, suggested that the localization delocalization edge is approached by mixing the non-magnetic 5*f* ^6^ Am ground state with the magnetic 5*f* ^7^ configuration, combined with hybridization with the 6*d* valence band (Savrasov *et al.*, 2006 ▸). The experiment, however, does not support such a scenario as no evidence of a mixed valence character emerges at high pressure (Heathman *et al.*, 2010 ▸).

RXES measurements at the *M*_4,5_ edges have the advantage over measurements at the *L* edge of interrogating directly 5*f* states. The lower energies involved (few keV), however, imply a reduced photon penetration depth (few hundreds of nm) and, therefore, attention must be paid to avoid any surface oxidation of the measured sample. Such experiments have been performed to elucidate the gradual conversion of the U oxidation state in mixed uranium oxides, with the direct observation of U(V) in a binary oxides (Kvashnina *et al.*, 2013 ▸; Gouder *et al.*, 2018 ▸), to study coordination complexes (Vitova *et al.*, 2010 ▸; Bès *et al.*, 2016 ▸), and uranium intermetallic compounds (Kvashnina *et al.*, 2017 ▸).

Kvashnina *et al.* (2017 ▸) report full core-to-core RXES maps for UPd_3_, USb, USn_3_, and URu_2_Si_2_. The experiment was performed at the ID26 beamline of ESRF by scanning the incident energy across the U *M*_4,5_ edges at different emission energies around the *M*_α_ and *M*_β_ emission lines. For UPd_3_, a localized system with 5*f* ^2^ configuration, incident energy scans at the maximum of the emission lines show a shift of the white line by ∼0.2 eV compared with UO_2_ (also a localized system with 5*f* ^2^ electron configuration but with an empty conduction band), and that the white line shift for 5*f* ^2^ (UPd_3_) and 5*f* ^3^ (USn_3_) in intermetallic compounds is smaller than in ionic compounds. For URu_2_Si_2_, where an itinerant character of the 5*f* electrons is expected, the spectral features observed at the *M* edges suggest almost tetravalent U atoms, with a 5*f* ^2^ configuration. This is in contrast with the analysis of *L*_3_ RXES data that gives a value *n*_*f*_ = 2.87 ± 0.08 for the 5*f* occupation number (Booth *et al.*, 2016 ▸), but in agreement with the conclusions of polarized neutron scattering (Santini *et al.*, 2000 ▸; Ressouche *et al.*, 2012 ▸), NIXS experiments (Sundermann *et al.*, 2016 ▸), and high-resolution RIXS measurements at the U *O* edges (Wray *et al.*, 2015 ▸). The discrepancy with the *L*_3_ RXES conclusions about *n*_*f*_ is probably due to 5*f*–6*d* hybridization effects that are more relevant at the *L*_3_ edge, where the 5*f* shell is interrogated only indirectly.

As written above, experiments at the *M* edges require clean surfaces, because of the small penetration depth of a few hundreds of nanometers. This requirement is much more stringent at the *N*_4,5_ edges (4*d* → 5*f*), where the penetration depth is of a few nanometers only. The reward for the efforts required is an energy resolution that can be as high as 30–35 meV, in an energy range up to 1 eV.

**Figure 15:**
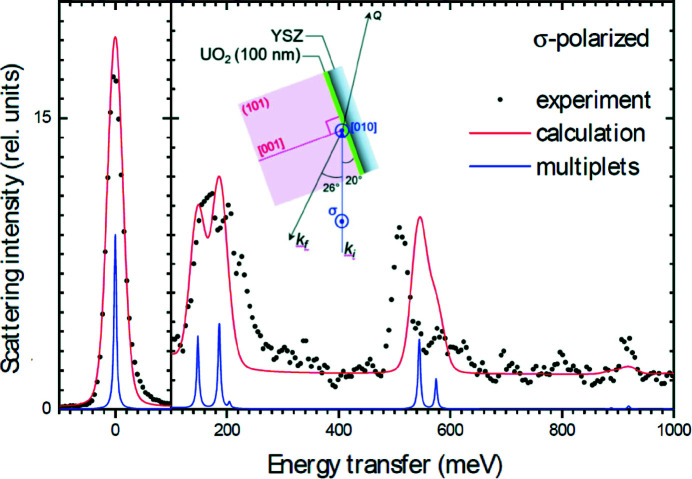
U *N*_4_-edge RIXS spectra of UO_2_ at *T* = 15 K. Data were taken with a photon energy of 778 eV for σ linear polarization, as shown in the inset. The red lines show the RIXS spectrum calculated for the U^4+^ 5*f* ^2^ configuration and CF parameters consistent with inelastic neutron scattering. The blue lines show the underlying multiplet peaks (with no line broadening) of the CF excitations around 180 meV and ^3^ *F*_2_ multiplet around 550 meV. Adapted from Lander *et al.* (2021 ▸).

Fig. 15 ▸ shows the 15-K RIXS spectrum at the uranium *N*_4_ absorption edge (778 eV), measured at the I21 beamline of the Diamond Light Source on atomically flat, epitaxial UO_2_ films (∼100 nm thickness) (Lander *et al.*, 2021 ▸). With a resolution of 35 meV, the crystal field excitations within the ^3^ *H*_4_ ground state multiplet are clearly resolved between ∼140 and 180 meV, confirming earlier inelastic neutron scattering (INS) studies (Amoretti *et al.*, 1989 ▸). INS measurements on UO_2_ failed to detect non-dipolar, higher-energy intermultiplet excitations. RIXS, instead, shows excitations at 520–580 meV due to transitions towards components of the ^3^ *F*_2_ excited multiplet. Measurements with π-polarization at the *N*_5_ edge (not shown in Fig. 15 ▸) also establish a peak at 920 meV due to a transition to the ^3^ *H*_5_ multiplet. RIXS experiments around the *O* edges (5*d* → 5*f*) have been attempted to determine the oxidation state of curium in oxide forms (Kvashnina *et al.*, 2007 ▸). At this edge, however, it is very complicated to access bulk properties by RIXS measurements.

## 8. High-resolution inelastic X-ray scattering

High-resolution inelastic X-ray scattering (IXS) at third-generation synchrotron radiation sources is a well established technique for mapping phonon branches with meV energy resolution. High-performance spectrometers using crystals in near-backscattering geometry and efficient focusing optics (Burkel, 2000 ▸) allow one to measure phonon dispersion curves in crystals with volumes as small as 10^−4^ mm^3^ and in epitaxial thin films less than 500 nm thick (d’Astuto *et al.*, 2002 ▸; Rennie *et al.*, 2018*a* ▸). Compared with neutrons, this is a crucial advantage for studying radioactive materials or, in general, systems for which large single crystals are not available, or high pressure limits the crystal size. The intrinsic background in IXS experiments is very low, the energy resolution is decoupled from energy transfer, and the momentum transfer is energy-independent. A drawback of the technique is that the scattering cross-section is proportional to the square of the atomic number, making it challenging to observe the contributions of light atoms to the vibrational spectra.

The ID28 beamline at ESRF is an example of a high-performance IXS spectrometer. In the incident energy range of interest for actinide systems (∼17–24 keV) a flat Si perfect crystal monochromator in back-scattering geometry, temperature controlled in the mK region, affords an energy resolution of about 1.5–3 meV when the analyzer is thermally stabilized to 6 × 10^−4^ K. Properly oriented single-crystal diamond slabs provide ideal windows if sample encapsulation is mandated by safety reasons.

**Figure 16:**
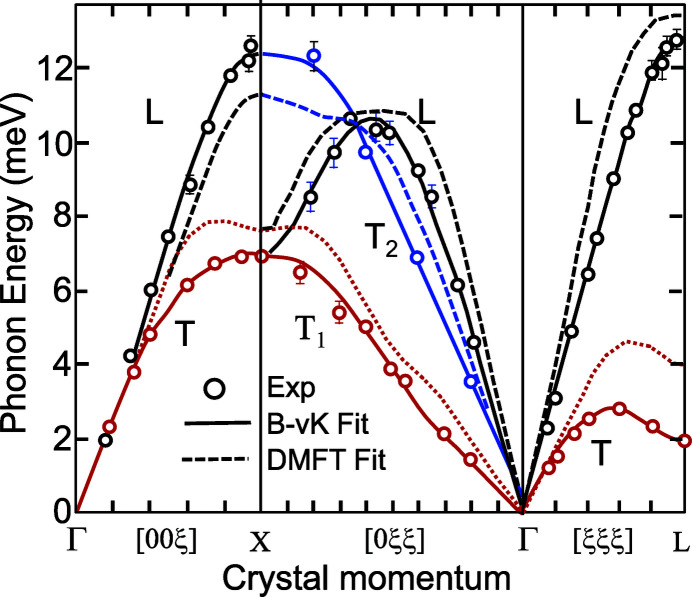
Phonon dispersion curves at *T* = 300 K along high-symmetry directions in δ-Pu stabilized by 0.6 wt% Ga alloying (*a* = 0.4621 nm). The experimental data are shown as circles [black longitudinal (L) modes; red and blue transverse (T) modes]. The branches T_1_ and T_2_ propagating along the [0ξξ] direction are polarized along 〈011〉 and 〈100〉, respectively. The solid curves are the fourth-nearest neighbor Born–von Kármán (B-vK) model fit. The dashed curves are calculated dispersions for pure δ-Pu based on dynamical mean field theory (DMFT) (Dai *et al.*, 2003 ▸). Adapted from Wong *et al.* (2003 ▸). Reprinted with permission from AAAS.

The measurements of the dispersion curves in f.c.c. δ-plutonium performed on large-grain polycrystalline samples are exemplary for illustrating the potential of IXS in the study of actinide materials (Wong *et al.*, 2003 ▸). The results, shown in Fig. 16 ▸, provide a qualitative validation of the predictions of dynamical mean field theory calculations (DMFT) (Dai *et al.*, 2003 ▸), showing that this theoretical approach is appropriate to describe not only the structure but also (at least qualitatively) the dynamics of strongly correlated electron systems. In particular, the experiment confirms the softening of the T[111] modes and the low-shear elastic modulus *C*′, reflecting the strong coupling between the lattice structure and the 5*f* valence instabilities.

IXS was also used to measure the phonon density of states of Ga-doped PuO_2_ (Manley *et al.*, 2012 ▸) and the phonon dispersion in NpO_2_, for which available single crystals are far too small for inelastic neutron scattering (Maldonado *et al.*, 2016 ▸).

The low-temperature properties of uranium metal were a mystery since elastic constant measurements in the 1960s found most unusual behaviors. The phonons were measured (Crummett *et al.*, 1979 ▸) with neutron inelastic scattering in 1979, showing unusual softening along the [100] direction of the orthorhombic structure of α-uranium. Following these measurements, a charge-density wave (CDW) was found to develop at 43 K (Lander *et al.*, 1994 ▸). α-U is the only element to exhibit such a CDW at ambient pressure. However, it was not until 2008 that a theory was presented for the phonons (Bouchet, 2008 ▸), and this immediately suggested that the phonon anomaly should be suppressed by pressure – a prediction confirmed by experiments (Raymond *et al.*, 2011 ▸) with ID28 at the ESRF using pressures up to 20 GPa. Later, experiments with thin films were successful in placing the [100] axis of α-U under tensile stress (Springell *et al.*, 2014 ▸), caused by interaction with the substrate, and the results were a CDW developing at 120 K, much higher temperature than in the bulk. The combination of theory and experiment showed the importance of the electron–phonon effects in the metal, and their momentum dependence. Properties such as the equation of state are affected by these considerations (Dewaele *et al.*, 2013 ▸).

Recently, the IXS spectrometer has been pushed to new limits by measuring both diffuse scattering and the phonon dispersion curves from a thin (300 nm) epitaxial film of U-Mo alloys. These materials have been of interest for many years, and might find applications as advanced nuclear fuels, but single crystals are not available and there has been controversy over whether the structures are body-centered cubic (b.c.c.) or something more complicated. Growing epitaxial films turned out to be relatively simple, and the subsequent diffuse scattering patterns (Chaney *et al.*, 2021 ▸) showed that the structure is essentially b.c.c. but superposed on that symmetry is correlated disorder, where the local symmetry is lower, as if the uranium atoms prefer to have neighbors reminiscent of the low symmetry found in the element at room temperature, and not the high symmetry demanded of b.c.c. The correlation length of such disorder depends on the composition, but is of the order of 5–10 nm.

**Figure 17:**
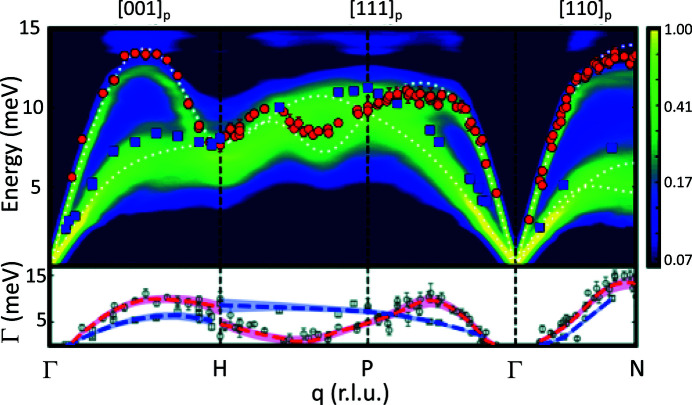
Phonon energy (top panel) and linewidth (bottom panel) dispersions on epitaxial U_1–*x*_Mo_*x*_ thin film alloys. Transverse (longitudinal) acoustic modes are shown as blue squares (red circles) for the alloy with 23 at% Mo. Theoretical results from a virtual crystal approximation for an alloy with 25 at% Mo are shown as dashed white lines. The full spectral function is plotted as a log_0.6_ color map to rescale the intensity divergence at gamma. All directions are within the parent BZ. The bottom panel shows raw linewidths, Γ_0_, as gray squares (TA) and circles (LA). Deconvoluted linewidths Γ_*d*_ are shown by dashed blue (TA) and red (LA) trend lines. Reprinted with permission from Chaney *et al.* (2021 ▸). Copyright (2021) by the American Physical Society.

The phonon dispersion curves, shown in Fig. 17 ▸, are close to those calculated by theory for this composition, except that they show large linewidths. The latter are much broader than expected for an alloy, and are due to the correlated disorder in the material. Thus, we see new effects in these materials that have been studied for many years.

In a similar vein, new experiments (Paolasini *et al.*, 2021 ▸) at ID28 on the phonon linewidths of UO_2_ below room temperature (this time using a single crystal rather than a thin epitaxial film) have shown that the linewidth broadening in UO_2_ is only along the [100] direction, and not in the other directions. Such anisotropic broadening has consequences for the thermal conductivity, which for a cubic material should not be anisotropic.

## 9. Conclusions

When the first synchrotrons began operations in the late 1970s and 1980s actinides were soon examined, notably with absorption (Kalkowski *et al.*, 1987*a* ▸; Bertram *et al.*, 1989 ▸) and EXAFS measurements (Silva & Nitsche, 1995 ▸; Conradson *et al.*, 1998 ▸) at the actinide *L* edges, high-pressure experiments using energy dispersive detectors (Benedict & Holzapfel, 1993 ▸), and high resolution XRD to investigate fine details of the diffraction patterns (Grübel *et al.*, 1991 ▸). The success of these machines across all parts of the periodic table led, of course, to more powerful synchrotrons being built, and the advantages for the study of actinides increased. They now represent some of the most powerful tools for such research.

This article has focused on experiments performed in the field of condensed-matter physics in the last 20 years at the ESRF in Grenoble, France, with some references to work at other synchrotrons, notably Diamond in the UK, and APS at Argonne National Laboratory (USA). Of course, many experiments have been performed in the domain of chemistry; efforts to study the dissolution of UO_2_ in water (Springell *et al.*, 2015 ▸; Rennie *et al.*, 2018*b* ▸) span these two fields. They demand synchrotron radiation, as diffraction signals are required from epitaxial films of <10 nm thickness. We are aware that studies of the physics and chemistry of actinides at the other two third-generation machines, ALS (USA) and SPring-8 (Japan), have been performed and new ones are also underway, and hope that some are covered in similar reviews in this journal.

In concluding this article, we would like to highlight four examples that we believe to have been ‘game changers’ for actinide research.

The first (Section 2) is the capability to observe samples at the µg level, allowing pressures up to 100 GPa to be applied in diamond-anvil cells, and the development of the angular-dependent data collection so sophisticated data analysis can be used to determine the crystallographic structures, see Fig. 2 ▸. These experiments, extended to transuranium samples (Lindbaum *et al.*, 2001 ▸; Heathman *et al.*, 2005 ▸), illustrated the failure of the density-functional theory (DFT) for actinides, and spurred the subsequent work with the dynamical mean-field theory (DMFT) that is frequently mentioned in the text. These experiments could only have been performed on third-generation synchrotrons.

The second example (Section 3) is the discovery in the actinide oxides and UPd_3_ of ordering of the electric quadrupolar moments (Santini *et al.*, 2009 ▸) below room temperature. In this case intensity is not always the main problem, but complex instrumentation is needed to measure the polarization dependence of the scattered photons, and the azimuthal dependence of the intensity. In the case of NpO_2_, for example, the nature of the phase transition at 25 K (Paixão *et al.*, 2002 ▸) had been the source of speculation for more than 50 years. How common this phenomenon is in the actinides is still an open question, but the tools to find such effects are now available.

The third example is that the development of the technique of XMCD (Section 4) led to a direct method of determining the absolute values of the spin and orbital moments in actinide materials. Prior to this, the only technique available was using polarized neutrons, but this requires sizable single crystals. Now these quantities can be measured with micrograms of polycrystalline samples. The values, and especially their ratio, are of major importance for actinide research, as with itinerant 5*f* electrons there is a tendency for a partial quenching of the orbital moment (Lander *et al.*, 1991 ▸). Now that the 〈*T*_*z*_〉 quantities calculated with intermediate coupling (see Fig. 10 ▸) can be used, the orbital to spin ratio is readily determined. Fig. 10 ▸ also represents perhaps the most convincing evidence given so far that intermediate coupling is a crucial requirement of the physics and chemistry of actinide materials in general.

The fourth example concerns the inelastic scattering of X-rays (Section 7). Here again, the capability to determine the phonon spectra from micrograms of material as at the ID28 beamline has been of great importance. The phonons of δ-Pu [Fig. 16 ▸ and Wong *et al.* (2003 ▸)] was a major achievement, especially since the theoretical work was actually published in advance (Dai *et al.*, 2003 ▸), and gave the first major ‘credibility test’ to the DMFT theory at that time (2003). More recently, the development of grazing-incidence IXS has led to important new information on the radiation damage in UO_2_ (Rennie *et al.*, 2018*a* ▸) and on the complexity of the U–Mo alloy system (Chaney *et al.*, 2021 ▸). The sensitivity of these experiments is outstanding; especially considering the sample mass is less than 100 µg.

The future of actinide research at synchrotrons is clearly a bright one. New machines, and associated complex instrumentation, are coming on-line that will clearly benefit new initiatives. Free-electron lasers (FELs) with pulse durations from a few to hundreds of femtoseconds are now starting to operate, pushing to the femtosecond range the timescale limits of spectroscopic and structure studies (Liermann *et al.*, 2021 ▸). Diffraction-limited storage rings (DLSRs) such as MAX-IV (Tavares *et al.*, 2018 ▸) and ESRF-EBS (Raimondi, 2016 ▸; Chenevier & Joly, 2020 ▸), with emittance approaching the diffraction limit and delivering ultra-small X-ray beams, start demonstrating their potential as an extraordinarily powerful tool for the investigation of complex systems and emerging phenomena. These machines will provide an increase in brightness and coherent flux of about two orders of magnitudes, compared with third-generation X-ray synchrotrons, with obvious applications for high pressure studies or coherent diffraction. All such instrumentation will greatly benefit our understanding of this complex row of elements in the periodic table.

## References

[bb125] Ament, L. J. P., van Veenendaal, M., Devereaux, T. P., Hill, J. P. & van den Brink, J. (2011). *Rev. Mod. Phys.* **83**, 705–767.

[bb143] Amoretti, G., Blaise, A., Caciuffo, R., Fournier, J. M., Hutchings, M. T., Osborn, R. & Taylor, A. D. (1989). *Phys. Rev. B*, **40**, 1856–1870. 10.1103/physrevb.40.1856 9992047

[bb146] Astuto, M. d’, Mang, P. K., Giura, P., Shukla, A., Ghigna, P., Mirone, A., Braden, M., Greven, M., Krisch, M. & Sette, F. (2002). *Phys. Rev. Lett.* **88**, 167002. 10.1103/PhysRevLett.88.167002 11955249

[bb65] Bao, Z., Springell, R., Walker, H. C., Leiste, H., Kuebel, K., Prang, R., Nisbet, G., Langridge, S., Ward, R. C. C., Gouder, T., Caciuffo, R. & Lander, G. H. (2013). *Phys. Rev. B*, **88**, 134426.

[bb160] Benedict, U. & Holzapfel, W. B. (1993). *Handbook of Physics and Chemistry of Rare Earths*, ch. 113, Vol. 17, edited by K. A. Gschneidner, L. Eyring, G. H. Lander and G. R. Choppin, p. 245. Amsterdam: North-Holland.

[bb64] Bernhoeft, N., Hiess, A., Langridge, S., Stunault, A., Wermeille, D., Vettier, C., Lander, G. H., Huth, M., Jourdan, M. & Adrian, H. (1998). *Phys. Rev. Lett.* **81**, 3419–3422.

[bb69] Bernhoeft, N., Lander, G. H., Longfield, M. J., Langridge, S., Mannix, D., Brown, S. D., Nuttall, W. J., Hiess, A., Vettier, C. & Lejay, P. (2004*b*). *J. Phys.: Condens. Matter*, **16**, 3869–3878.

[bb63] Bernhoeft, N., Paixão, J. A., Detlefs, C., Wilkins, S. B., Javorský, P., Blackburn, E. & Lander, G. H. (2004*a*). *Phys. Rev. B*, **69**, 174415.

[bb72] Bertram, S., Kaindl, G., Jové, J., Pagès, M. & Gal, J. (1989). *Phys. Rev. Lett.* **63**, 2680–2683. 10.1103/PhysRevLett.63.2680 10040959

[bb138] Bès, R., Rivenet, M., Solari, P.-L., Kvashnina, K. O., Scheinost, A. C. & Martin, P. M. (2016). *Inorg. Chem.* **55**, 4260–4270. 10.1021/acs.inorgchem.6b00014 27132487

[bb40] Blackburn, E., Caciuffo, R., Magnani, N., Santini, P., Brown, P. J., Enderle, M. & Lander, G. H. (2005). *Phys. Rev. B*, **72**, 184411.

[bb128] Booth, C., Jiang, Y., Wang, D., Mitchell, J., Tobash, P., Bauer, E., Wall, M., Allen, P., Sokaras, D., Nordlund, D., Weng, T.-C., Torrez, M. & Sarrao, J. L. (2012). *Proc. Natl Acad. Sci. USA*, **109**, 10205–10209. 10.1073/pnas.1200725109 PMC3387120 22706643

[bb129] Booth, C. H., Medling, S. A., Tobin, J. G., Baumbach, R. E., Bauer, E. D., Sokaras, D., Nordlund, D. & Weng, T. (2016). *Phys. Rev. B*, **94**, 045121.

[bb154] Bouchet, J. (2008). *Phys. Rev. B*, **77**, 024113.

[bb113] Bradley, J. A., Sen Gupta, S., Seidler, G. T., Moore, K. T., Haverkort, M. W., Sawatzky, G. A., Conradson, S. D., Clark, D. L., Kozimor, S. A. & Boland, K. S. (2010). *Phys. Rev. B*, **81**, 193104.

[bb123] Braicovich, L., van den Brink, J., Bisogni, V., Sala, M. M., Ament, L. J. P., Brookes, N. B., De Luca, G. M., Salluzzo, M., Schmitt, T., Strocov, V. N. & Ghiringhelli, G. (2010). *Phys. Rev. Lett.* **104**, 077002. 10.1103/PhysRevLett.104.077002 20366909

[bb46] Broholm, C., Lin, H., Matthews, P. T., Mason, T. E., Buyers, W. J. L., Collins, M. F., Menovsky, A. A., Mydosh, J. A. & Kjems, J. K. (1991). *Phys. Rev. B*, **43**, 12809–12822. 10.1103/physrevb.43.12809 9997095

[bb145] Burkel, E. (2000). *Rep. Prog. Phys.* **63**, 171–232.

[bb39] Burlet, P., Rossat-Mignod, J., vuevel, S., Vogt, O., Spirlet, J. C. & Rebivant, J. (1986). *J. Less-Common Met.* **121**, 121–139.

[bb84] Caciuffo, R., Buck, E. C., Clark, D. L. & van der Laan, G. (2010*a*). *MRS Bull.* **35**, 889–895.

[bb42] Caciuffo, R., Lander, G. H., Spirlet, J. C., Fournier, J. M. & Kuhs, W. F. (1987). *Solid State Commun.* **64**, 149–152.

[bb37] Caciuffo, R., Paix o, J. A., Detlefs, C., Longfield, M. J., Santini, P., Bernhoeft, N., Rebizant, J. & Lander, G. H. (2003). *J. Phys. Condens. Matter*, **15**, S2287–S2296. 10.1103/PhysRevLett.89.187202 12398632

[bb112] Caciuffo, R., van der Laan, G., Simonelli, L., Vitova, T., Mazzoli, C., Denecke, M. A. & Lander, G. H. (2010*b*). *Phys. Rev. B*, **81**, 195104.

[bb93] Carra, P., Thole, B. T., Altarelli, M. & Wang, X. (1993). *Phys. Rev. Lett.* **70**, 694–697. 10.1103/PhysRevLett.70.694 10054179

[bb12] Carter, K. P., Shield, K. M., Smith, K. F., Jones, Z. R., Wacker, J. N., Arnedo-Sanchez, L., Mattox, T. M., Moreau, L. M., Knope, K. E., Kozimor, S. A., Booth, C. H. & Abergel, R. J. (2021). *Nature*, **590**, 85–88. 10.1038/s41586-020-03179-3 33536647

[bb53] Chandra, P., Coleman, P. & Flint, R. (2015). *Phys. Rev. B*, **91**, 205103.

[bb158] Chaney, D., Castellano, A., Bosak, A., Bouchet, J., Bottin, F., Dorado, B., Paolasini, L., Rennie, S., Bell, C., Springell, R. & Lander, G. H. (2021). *Phys. Rev. Mater.* **5**, 035004.

[bb168] Chenevier, D. & Joly, A. (2020). *Synchrotron Radiat. News*, **33**(3), 37–41.

[bb80] Clark, D. R., Janecky, D. L. & Lane, L. J. (2006). *Phys. Today*, **59**, 34–40.

[bb95] Collins, S. P., Laundy, D., Tang, C. C. & Laan, G. (1995). *J. Phys. Condens. Matter*, **7**, 9325–9341.

[bb75] Conradson, S. D., Al Mahamid, I., Clark, D. L., Hess, N. J., Hudson, E. A., Neu, M. P., Palmer, P. D., Runde, W. H. & Drew Tait, C. (1998). *Polyhedron*, **17**, 599–602.

[bb152] Crummett, W. P., Smith, H. G., Nicklow, R. M. & Wakabayashi, N. (1979). *Phys. Rev. B*, **19**, 6028–6037.

[bb15] Daghero, D., Tortello, M., Ummarino, G. A., Griveau, J.-C., Colineau, E., Eloirdi, R., Shick, A. B., Kolorenc, J., Lichtenstein, A. I. & Caciuffo, R. (2012). *Nat. Commun.* **3**, 786. 10.1038/ncomms1785 PMC3337991 22510691

[bb149] Dai, X., Savrasov, S. Y., Kotliar, G., Migliori, A., Ledbetter, H. & Abrahams, E. (2003). *Science*, **300**, 953–955. 10.1126/science.1083428 12738856

[bb77] Dardenne, K., Brendebach, B., Denecke, M. A., Liu, X., Rothe, J. & Vitova, T. (2009). *J. Phys. Conf. Ser.* **190**, 012037.

[bb102] Das, T., Zhu, J.-X. & Graf, M. J. (2015). *Sci. Rep.* **5**, 8632. 10.1038/srep08632 PMC4342998 25721375

[bb76] Denecke, M. A. (2006). *Coord. Chem. Rev.* **250**, 730–754.

[bb157] Dewaele, A., Bouchet, J., Occelli, F., Hanfland, M. & Garbarino, G. (2013). *Phys. Rev. B*, **88**, 134202.

[bb87] Dieste, O., Wiss, T., Griveau, J.-C., Konings, R. J. M., van der Laan, G. & Caciuffo, R. (2019). *Mater. Res. Expr.* **6**, 026307.

[bb20] Dudarev, S. L., Liu, P., Andersson, D. A., Stanek, C. R., Ozaki, T. & Franchini, C. (2019). *Phys. Rev. Mater.* **3**, 083802.

[bb25] Eloirdi, R., Giacobbe, C., Celdran, P. A., Magnani, N., Lander, G. H., Griveau, J.-C., Colineau, E., Miyake, K. & Caciuffo, R. (2017). *Phys. Rev. B*, **95**, 094517.

[bb41] Faber, J., Lander, G. H. & Cooper, B. R. (1975). *Phys. Rev. Lett.* **35**, 1770–1773.

[bb122] Ghiringhelli, G., Brookes, N. B., Annese, E., Berger, H., Dallera, C., Grioni, M., Perfetti, L., Tagliaferri, A. & Braicovich, L. (2004). *Phys. Rev. Lett.* **92**, 117406. 10.1103/PhysRevLett.92.117406 15089169

[bb31] Gibbs, D., Harshman, D. R., Isaacs, E. D., McWhan, D. B., Mills, D. & Vettier, C. (1988). *Phys. Rev. Lett.* **61**, 1241–1244. 10.1103/PhysRevLett.61.1241 10038738

[bb110] Gordon, R. A., Seidler, G. T., Fister, T. T., Haverkort, M. W., Sawatzky, G. A., Tanaka, A. & Sham, T. K. (2008). *Europhys. Lett.* **81**, 26004.

[bb137] Gouder, T., Eloirdi, R. & Caciuffo, R. (2018). *Sci. Rep.* **8**, 8306. 10.1038/s41598-018-26594-z PMC5974404 29844333

[bb89] Goulon, J., Goulon-Ginet, C., Cortes, R. & Dubois, J. M. (1982). *J. Phys. Fr.* **43**, 539–548.

[bb134] Griveau, J.-C., Rebizant, J., Lander, G. H. & Kotliar, G. (2005). *Phys. Rev. Lett.* **94**, 097002. 10.1103/PhysRevLett.94.097002 15783989

[bb161] Grübel, G., Axe, J. D., Gibbs, D., Lander, G. H., Marmeggi, J. C. & Brückel, T. (1991). *Phys. Rev. B*, **43**, 8803–8807. 10.1103/physrevb.43.8803 9996547

[bb105] Gurtubay, I. G., Pitarke, J. M., Ku, W., Eguiluz, A. G., Larson, B. C., Tischler, J., Zschack, P. & Finkelstein, K. D. (2005). *Phys. Rev. B*, **72**, 125117.

[bb29] Hannon, J., Trammell, G., Blume, M. & Gibbs, D. (1988). *Phys. Rev. Lett.* **61**, 1245–1248. 10.1103/PhysRevLett.61.1245 10038739

[bb30] Hannon, J., Trammell, G., Blume, M. & Gibbs, D. (1989). *Phys. Rev. Lett.* **62**, 2644. 10.1103/PhysRevLett.61.1245 10038739

[bb107] Haverkort, M. W., Tanaka, A., Tjeng, L. H. & Sawatzky, G. A. (2007). *Phys. Rev. Lett.* **99**, 257401. 10.1103/PhysRevLett.99.257401 18233556

[bb10] Heathman, S., Haire, R. G., Le Bihan, T., Lindbaum, A., Idiri, M., Normile, P., Li, S., Ahuja, R., Johansson, B. & Lander, G. H. (2005). *Science*, **309**, 110–113. 10.1126/science.1112453 15994550

[bb133] Heathman, S., Haire, R. G., Le Bihan, T., Lindbaum, A., Litfin, K., Méresse, Y. & Libotte, H. (2000). *Phys. Rev. Lett.* **85**, 2961–2964. 10.1103/PhysRevLett.85.2961 11005978

[bb11] Heathman, S., Le Bihan, T., Yagoubi, S., Johansson, B. & Ahuja, R. (2013). *Phys. Rev. B*, **87**, 214111.

[bb132] Heathman, S., Rueff, J.-P., Simonelli, L., Denecke, M. A., Griveau, J.-C., Caciuffo, R. & Lander, G. H. (2010). *Phys. Rev. B*, **82**, 201103.

[bb103] Hiess, A., Stunault, A., Colineau, E., Rebizant, J., Wastin, F., Caciuffo, R. & Lander, G. H. (2008). *Phys. Rev. Lett.* **100**, 076403. 10.1103/PhysRevLett.100.076403 18352577

[bb33] Hill, J. P. & McMorrow, D. F. (1996). *Acta Cryst.* A**52**, 236–244.

[bb27] Hiranaka, Y., Nakamura, A., Hedo, M., Takeuchi, T., Mori, A., Hirose, Y., Mitamura, K., Sugiyama, K., Hagiwara, M., Nakama, T. & Ōnuki, Y. (2013). *J. Phys. Soc. Jpn*, **82**, 083708.

[bb32] Isaacs, E. D., McWhan, D. B., Peters, C., Ice, G. E., Siddons, D. P., Hastings, J. B., Vettier, C. & Vogt, O. (1989). *Phys. Rev. Lett.* **62**, 1671–1674. 10.1103/PhysRevLett.62.1671 10039734

[bb99] Janoschek, M., Haskel, D., Fernandez-Rodriguez, J., van Veenendaal, M., Rebizant, J., Lander, G. H., Zhu, J.-X., Thompson, J. D. & Bauer, E. D. (2015). *Phys. Rev. B*, **91**, 035117.

[bb14] Joyce, J. J. & Lander, G. H. (2019). *Plutonium Handbook*, ch. 15, edited by D. L. Clark, D. A. Geeson and R. J. Hanrahan, p. 1203. Oak Brook: American Nuclear Society.

[bb101] Jutier, F., Ummarino, G. A., Griveau, J.-C., Wastin, F., Colineau, E., Rebizant, J., Magnani, N. & Caciuffo, R. (2008). *Phys. Rev. B*, **77**, 024521.

[bb71] Kalkowski, G., Kaindl, G., Bertram, S., Schmiester, G., Rebizant, J., Spirlet, J. C. & Vogt, O. (1987*a*). *Solid State Commun.* **64**, 193–196.

[bb73] Kalkowski, G., Kaindl, G., Brewer, W. D. & Krone, W. (1987*b*). *Phys. Rev. B*, **35**, 2667–2677. 10.1103/physrevb.35.2667 9941740

[bb121] Kotani, A. & Shin, S. (2001). *Rev. Mod. Phys.* **73**, 203–246.

[bb136] Kvashnina, K. O., Butorin, S. M., Martin, P. & Glatzel, P. (2013). *Phys. Rev. Lett.* **111**, 253002. 10.1103/PhysRevLett.111.253002 24483742

[bb144] Kvashnina, K. O., Butorin, S. M., Shuh, D. K., Guo, J.-H., Werme, L. & Nordgren, J. (2007). *Phys. Rev. B*, **75**, 115107.

[bb127] Kvashnina, K. O., Kvashnin, Y. O. & Butorin, S. M. (2014). *J. Electron Spectrosc. Relat. Phenom.* **194**, 27–36.

[bb139] Kvashnina, K. O., Walker, H. C., Magnani, N., Lander, G. H. & Caciuffo, R. (2017). *Phys. Rev. B*, **95**, 245103.

[bb88] Laan, G. (2013). *J. Phys. Conf. Ser.* **430**, 012127.

[bb109] Laan, G. van der (2012*a*). *Phys. Rev. B*, **86**, 035138.

[bb120] Laan, G. van der (2012*b*). *Phys. Rev. Lett.* **108**, 077401. 10.1103/PhysRevLett.108.077401 22401253

[bb82] Laan, G. van der, Moore, K. T., Tobin, J. G., Chung, B. W., Wall, M. A. & Schwartz, A. J. (2004). *Phys. Rev. Lett.* **93**, 097401. 10.1103/PhysRevLett.93.097401 15447136

[bb94] Laan, G. van der & Thole, B. T. (1996). *Phys. Rev. B*, **53**, 14458–14469. 10.1103/physrevb.53.14458 9983244

[bb153] Lander, G., Fisher, E. & Bader, S. D. (1994). *Adv. Phys.* **43**, 1–111.

[bb164] Lander, G. H., Brooks, M. S. S. & Johansson, B. (1991). *Phys. Rev. B*, **43**, 13672–13675. 10.1103/physrevb.43.13672 9997214

[bb96] Lander, G. H., Griveau, J.-C., Eloirdi, R., Magnani, N., Colineau, E., Wilhelm, F., Brown, S. D., Wermeille, D., Rogalev, A., Haire, R. G. & Caciuffo, R. (2019). *Phys. Rev. B*, **99**, 224419.

[bb140] Lander, G. H., Sundermann, M., Springell, R., Walters, A. C., Nag, A., Garcia-Fernandez, M., Zhou, K. J., van der Laan, G. & Caciuffo, R. (2021). *J. Phys. Condens. Matter*, **33**, 06LT01. 10.1088/1361-648X/abc4d2 33325375

[bb57] Langridge, S., Stirling, W. G., Lander, G. H. & Rebizant, J. (1994*a*). *Phys. Rev. B*, **49**, 12010–12021. 10.1103/physrevb.49.12010 10010072

[bb58] Langridge, S., Stirling, W. G., Lander, G. H. & Rebizant, J. (1994*b*). *Phys. Rev. B*, **49**, 12022–12029. 10.1103/physrevb.49.12022 10010073

[bb68] Langridge, S., Watson, G. M., Gibbs, D., Betouras, J. J., Gidopoulos, N. I., Pollmann, F., Long, M. W., Vettier, C. & Lander, G. H. (2014). *Phys. Rev. Lett.* **112**, 167201. 10.1103/PhysRevLett.112.167201 24815664

[bb106] Larson, B. C., Ku, W., Tischler, J. Z., Lee, C.-C., Restrepo, O. D., Eguiluz, A. G., Zschack, P. & Finkelstein, K. D. (2007). *Phys. Rev. Lett.* **99**, 026401. 10.1103/PhysRevLett.99.026401 17678238

[bb9] Lashley, J. C. & Lawson, A. (2019). *Plutonium Handbook*, ch. 7, edited by D. L. Clark, D. A. Geeson and R. J. Hanrahan, p. 287. Oak Brook: American Nuclear Society.

[bb13] Lashley, J. C., Lawson, A., McQueeney, R. J. & Lander, G. H. (2005). *Phys. Rev. B*, **72**, 054416.

[bb56] Lawrence Bright, E., Springell, R., Porter, D. G., Collins, S. P. & Lander, G. H. (2019). *Phys. Rev. B*, **100**, 134426.

[bb23] Le Bihan, T., Heathman, S., Idiri, M., Lander, G. H., Wills, J. M., Lawson, A. C. & Lindbaum, A. (2003). *Phys. Rev. B*, **67**, 134102.

[bb61] Lidström, E., Mannix, D., Hiess, A., Rebizant, J., Wastin, F., Lander, G. H., Marri, I., Carra, P., Vettier, C. & Longfield, M. J. (2000). *Phys. Rev. B*, **61**, 1375–1385.

[bb165] Liermann, H. P., Konôpková, Z., Appel, K., Prescher, C., Schropp, A., Cerantola, V., Husband, R. J., McHardy, J. D., McMahon, M. I., McWilliams, R. S., Pépin, C. M., Mainberger, J., Roeper, M., Berghäuser, A., Damker, H., Talkovski, P., Foese, M., Kujala, N., Ball, O. B., Baron, M. A., Briggs, R., Bykov, M., Bykova, E., Chantel, J., Coleman, A. L., Cynn, H., Dattelbaum, D., Dresselhaus-Marais, L. E., Eggert, J. H., Ehm, L., Evans, W. J., Fiquet, G., Frost, M., Glazyrin, K., Goncharov, A. F., Hwang, H., Jenei, Z., Kim, J.-Y., Langenhorst, F., Lee, Y., Makita, M., Marquardt, H., McBride, E. E., Merkel, S., Morard, G., O’Bannon, E. F., Otzen, C., Pace, E. J., Pelka, A., Pigott, J. S., Prakapenka, V. B., Redmer, R., Sanchez-Valle, C., Schoelmerich, M., Speziale, S., Spiekermann, G., Sturtevant, B. T., Toleikis, S., Velisavljevic, N., Wilke, M., Yoo, C.-S., Baehtz, C., Zastrau, U. & Strohm, C. (2021). *J. Synchrotron Rad.* **28**, 688–706. 10.1107/S1600577521002551 PMC8127375 33949979

[bb24] Lindbaum, A., Heathman, S., Litfin, K., Méresse, Y., Haire, R. G., Le Bihan, T. & Libotte, H. (2001). *Phys. Rev. B*, **63**, 214101. 10.1103/PhysRevLett.85.2961 11005978

[bb62] Longfield, M. J., Paixão, J. A., Bernhoeft, N. & Lander, G. H. (2002). *Phys. Rev. B*, **66**, 054417. 10.1103/PhysRevLett.89.187202 12398632

[bb104] Macrander, A. T., Montano, P. A., Price, D. L., Kushnir, V. I., Blasdell, R. C., Kao, C. C. & Cooper, B. R. (1996). *Phys. Rev. B*, **54**, 305–312. 10.1103/physrevb.54.305 9984259

[bb97] Magnani, N., Caciuffo, R., Wilhelm, F., Colineau, E., Eloirdi, R., Griveau, J.-C., Rusz, J., Oppeneer, P. M., Rogalev, A. & Lander, G. H. (2015). *Phys. Rev. Lett.* **114**, 097203. 10.1103/PhysRevLett.114.097203 25793847

[bb44] Magnani, N., Carretta, S., Caciuffo, R., Santini, P., Amoretti, G., Hiess, A., Rebizant, J. & Lander, G. H. (2008). *Phys. Rev. B*, **78**, 104425.

[bb100] Magnani, N., Eloirdi, R., Wilhelm, F., Colineau, E., Griveau, J.-C., Shick, A. B., Lander, G. H., Rogalev, A. & Caciuffo, R. (2017). *Phys. Rev. Lett.* **119**, 157204. 10.1103/PhysRevLett.119.157204 29077471

[bb151] Maldonado, P., Paolasini, L., Oppeneer, P. M., Forrest, T. R., Prodi, A., Magnani, N., Bosak, A., Lander, G. H. & Caciuffo, R. (2016). *Phys. Rev. B*, **93**, 144301.

[bb150] Manley, M. E., Jeffries, J. R., Said, A. H., Marianetti, C. A., Cynn, H., Leu, B. M. & Wall, M. A. (2012). *Phys. Rev. B*, **85**, 132301.

[bb47] Mason, T. E., Gaulin, B. D., Garrett, J. D., Tun, Z., Buyers, W. J. L. & Isaacs, E. D. (1990). *Phys. Rev. Lett.* **65**, 3189–3192. 10.1103/PhysRevLett.65.3189 10042804

[bb54] McMorrow, D. F., McEwen, K. A., Steigenberger, U., Rønnow, H. M. & Yakhou, F. (2001). *Phys. Rev. Lett.* **87**, 057201. 10.1103/PhysRevLett.87.057201 11497802

[bb28] Miyake, S. & Watanabe, S. (2014). *J. Phys. Soc. Jpn*, **83**, 061006.

[bb3] Moore, K. T. & van der Laan, G. (2009). *Rev. Mod. Phys.* **81**, 235–298.

[bb85] Moore, K. T., van der Laan, G., Haire, R. G., Wall, M. A. & Schwartz, A. J. (2006). *Phys. Rev. B*, **73**, 033109. 10.1103/PhysRevLett.98.236402 17677923

[bb86] Moore, K. T., van der Laan, G., Haire, R. G., Wall, M. A., Schwartz, A. J. & Söderlind, P. (2007). *Phys. Rev. Lett.* **98**, 236402. 10.1103/PhysRevLett.98.236402 17677923

[bb2] Mydosh, J. A. & Oppeneer, P. M. (2011). *Rev. Mod. Phys.* **83**, 1301–1322.

[bb45] Mydosh, J. A., Oppeneer, P. M. & Riseborough, P. S. (2020). *J. Phys. Condens. Matter*, **32**, 143002. 10.1088/1361-648X/ab5eba 31801118

[bb34] Nagao, T. & Igarashi, J. (2005). *Phys. Rev. B*, **72**, 174421.

[bb59] Normile, P. S., Stirling, W. G., Mannix, D., Lander, G. H., Wastin, F., Rebizant, J., Boudarot, F., Burlet, P., Lebech, B. & Coburn, S. (2002*a*). *Phys. Rev. B*, **66**, 014405.

[bb60] Normile, P. S., Stirling, W. G., Mannix, D., Lander, G. H., Wastin, F., Rebizant, J. & Coburn, S. (2002*b*). *Phys. Rev. B*, **66**, 014406.

[bb49] Oppeneer, P. M., Elgazzar, S., Rusz, J., Feng, Q., Durakiewicz, T. & Mydosh, J. A. (2011). *Phys. Rev. B*, **84**, 241102.

[bb36] Paixão, J. A., Detlefs, C., Longfield, M. J., Caciuffo, R., Santini, P., Bernhoeft, N., Rebizant, J. & Lander, G. H. (2002). *Phys. Rev. Lett.* **89**, 187202. 10.1103/PhysRevLett.89.187202 12398632

[bb159] Paolasini, L., Chaney, D., Bosak, A., Lander, G. H. & Caciuffo, R. (2021). *Phys. Rev. B*, **104**, 024305.

[bb19] Pezzoli, M. E., Haule, K. & Kotliar, G. (2011). *Phys. Rev. Lett.* **106**, 016403. 10.1103/PhysRevLett.106.016403 21231758

[bb91] Pfalzer, P., Urbach, J.-P., Klemm, M., Horn, S., denBoer, M. L., Frenkel, A. I. & Kirkland, J. P. (1999). *Phys. Rev. B*, **60**, 9335–9339.

[bb21] Pourovskii, L. V. & Khmelevskyi, S. (2021). *Proc. Natl Acad. Sci. USA*, **118**, e2025317118. 10.1073/pnas.2025317118 PMC8040619 33795518

[bb70] Prokeš, K., Lander, G. H. & Bernhoeft, N. (2009). *J. Phys. Condens. Matter*, **21**, 285402. 10.1088/0953-8984/21/28/285402 21828520

[bb167] Raimondi, P. (2016). *Synchrotron Radiat. News*, **29**(6), 8–15.

[bb26] Ramshaw, B. J., Shekhter, A., McDonald, R. D., Betts, J. B., Mitchell, J. N., Tobash, P. H., Mielke, C. H., Bauer, E. D. & Migliori, A. (2015). *Proc. Natl Acad. Sci. USA*, **112**, 3285–3289. 10.1073/pnas.1421174112 PMC4371925 25737548

[bb155] Raymond, S., Bouchet, J., Lander, G. H., Le Tacon, M., Garbarino, G., Hoesch, M., Rueff, J.-P., Krisch, M., Lashley, J. C., Schulze, R. K. & Albers, R. C. (2011). *Phys. Rev. Lett.* **107**, 136401. 10.1103/PhysRevLett.107.136401 22026877

[bb147] Rennie, S., Lawrence Bright, E., Darnbrough, J. E., Paolasini, L., Bosak, A., Smith, A. D., Mason, N., Lander, G. H. & Springell, R. (2018*a*). *Phys. Rev. B*, **97**, 224303.

[bb163] Rennie, S., Lawrence Bright, E., Sutcliffe, J. E., Darnbrough, J. E., Burrows, R., Rawle, J., Nicklin, C., Lander, G. H. & Springell, R. (2018*b*). *Corros. Sci.* **145**, 162–169.

[bb141] Ressouche, E., Ballou, R., Bourdarot, F., Aoki, D., Simonet, V., Fernandez-Diaz, M. T., Stunault, A. & Flouquet, J. (2012). *Phys. Rev. Lett.* **109**, 067202. 10.1103/PhysRevLett.109.067202 23006299

[bb131] Rueff, J.-P., Raymond, S., Yaresko, A., Braithwaite, D., Leininger, P., Vankó, G., Huxley, A., Rebizant, J. & Sato, N. (2007). *Phys. Rev. B*, **76**, 085113.

[bb124] Rueff, J.-P. & Shukla, A. (2010). *Rev. Mod. Phys.* **82**, 847–896.

[bb50] Santini, P. & Amoretti, G. (1994). *Phys. Rev. Lett.* **73**, 1027–1030. 10.1103/PhysRevLett.73.1027 10057601

[bb51] Santini, P., Amoretti, G., Caciuffo, R., Bourdarot, F. & Fåk, B. (2000). *Phys. Rev. Lett.* **85**, 654–657. 10.1103/PhysRevLett.85.654 10991363

[bb4] Santini, P., Carretta, S., Amoretti, G., Caciuffo, R., Magnani, N. & Lander, G. H. (2009). *Rev. Mod. Phys.* **81**, 807–863.

[bb43] Santini, P., Carretta, S., Magnani, N., Amoretti, G. & Caciuffo, R. (2006). *Phys. Rev. Lett.* **97**, 207203. 10.1103/PhysRevLett.97.207203 17155710

[bb1] Sarrao, J. L., Morales, L. A., Thompson, J. D., Scott, B. L., Stewart, G. R., Wastin, F., Rebizant, J., Boulet, P., Colineau, E. & Lander, G. H. (2002). *Nature*, **420**, 297–299. 10.1038/nature01212 12447434

[bb135] Savrasov, S. Y., Haule, K. & Kotliar, G. (2006). *Phys. Rev. Lett.* **96**, 036404. 10.1103/PhysRevLett.96.036404 16486744

[bb79] Scheinost, A. C., Claussner, J., Exner, J., Feig, M., Findeisen, S., Hennig, C., Kvashnina, K. O., Naudet, D., Prieur, D., Rossberg, A., Schmidt, M., Qiu, C., Colomp, P., Cohen, C., Dettona, E., Dyadkin, V. & Stumpf, T. (2021). *J. Synchrotron Rad.* **28**, 333–349. 10.1107/S1600577520014265 PMC7842221 33399586

[bb111] Schülke, W. (2007). *Electron Dynamics by Inelastic X-ray Scattering.* Oxford: Oxford University Press.

[bb114] Sen Gupta, S., Bradley, J. A., Haverkort, M. W., Seidler, G. T., Tanaka, A. & Sawatzky, G. A. (2011). *Phys. Rev. B*, **84**, 075134.

[bb22] Shick, A. B., Fujimori, S. & Pickett, W. E. (2021). *Phys. Rev. B*, **103**, 125136.

[bb16] Shick, A. B., Kolorenc, J., Rusz, J., Oppeneer, P. M., Lichtenstein, A. I., Katsnelson, M. I. & Caciuffo, R. (2013). *Phys. Rev. B*, **87**, 020505.

[bb17] Shim, J., Haule, K. & Kotliar, G. (2007). *Nature*, **446**, 513–516. 10.1038/nature05647 17392780

[bb83] Shim, J. H., Haule, K. & Kotliar, G. (2009). *Europhys. Lett.* **85**, 17007.

[bb74] Silva, R. J. & Nitsche, H. (1995). *Radiochim. Acta*, **70–71**, 377–396.

[bb162] Springell, R., Rennie, S., Costelle, L., Darnbrough, J., Stitt, C., Cocklin, E., Lucas, C., Burrows, R., Sims, H., Wermeille, D., Rawle, J., Nicklin, C., Nuttall, W., Scott, T. & Lander, G. (2015). *Faraday Discuss.* **180**, 301–311. 10.1039/c4fd00254g 25932469

[bb156] Springell, R., Ward, R. C. C., Bouchet, J., Chivall, J., Wermeille, D., Normile, P. S., Langridge, S., Zochowski, S. W. & Lander, G. H. (2014). *Phys. Rev. B*, **89**, 245101.

[bb115] Sundermann, M., Haverkort, M. W., Agrestini, S., Al-Zein, A., Moretti Sala, M., Huang, Y., Golden, M., de Visser, A., Thalmeier, P., Tjeng, L. H. & Severing, A. (2016). *Proc. Natl Acad. Sci. USA*, **113**, 13989–13994. 10.1073/pnas.1612791113 PMC5150412 27872287

[bb117] Sundermann, M., Simonelli, L., Huotari, S., Eloirdi, R., Lander, G. H., Caciuffo, R. & van der Laan, G. (2020). *Phys. Rev. B*, **101**, 075103.

[bb116] Sundermann, M., van der Laan, G., Severing, A., Simonelli, L., Lander, G. H., Haverkort, M. W. & Caciuffo, R. (2018). *Phys. Rev. B*, **98**, 205108.

[bb18] Suzuki, M.-T., Magnani, N. & Oppeneer, P. M. (2010). *Phys. Rev. B*, **82**, 241103.

[bb166] Tavares, P. F., Al-Dmour, E., Andersson, Å., Cullinan, F., Jensen, B. N., Olsson, D., Olsson, D. K., Sjöström, M., Tarawneh, H., Thorin, S. & Vorozhtsov, A. (2018). *J. Synchrotron Rad.* **25**, 1291–1316. 10.1107/S1600577518008111 PMC6140400 30179168

[bb92] Thole, B. T., Carra, P., Sette, F. & van der Laan, G. (1992). *Phys. Rev. Lett.* **68**, 1943–1946. 10.1103/PhysRevLett.68.1943 10045260

[bb81] Thole, B. T. & van der Laan, G. (1988). *Phys. Rev. B*, **38**, 3158–3171. 10.1103/physrevb.38.3158 9946656

[bb130] Tobin, J. G., Yu, S.-W., Booth, C. H., Tyliszczak, T., Shuh, D. K., van der Laan, G., Sokaras, D., Nordlund, D., Weng, T.-C. & Bagus, P. S. (2015). *Phys. Rev. B*, **92**, 035111.

[bb90] Tröger, L., Arvanitis, D., Baberschke, K., Michaelis, H., Grimm, U. & Zschech, E. (1992). *Phys. Rev. B*, **46**, 3283–3289. 10.1103/physrevb.46.3283 10004043

[bb108] Veenendaal, M. van & Haverkort, M. W. (2008). *Phys. Rev. B*, **77**, 224107.

[bb118] Verbeni, R., Pylkkänen, T., Huotari, S., Simonelli, L., Vankó, G., Martel, K., Henriquet, C. & Monaco, G. (2009). *J. Synchrotron Rad.* **16**, 469–476. 10.1107/S090904950901886X 19535859

[bb126] Vitova, T., Kvashnina, K. O., Nocton, G., Sukharina, G., Denecke, M. A., Butorin, S. M., Mazzanti, M., Caciuffo, R., Soldatov, A., Behrends, T. & Geckeis, H. (2010). *Phys. Rev. B*, **82**, 235118.

[bb52] Walker, H. C., Caciuffo, R., Aoki, D., Bourdarot, F., Lander, G. H. & Flouquet, J. (2011). *Phys. Rev. B*, **83**, 193102.

[bb55] Walker, H. C., McEwen, K. A., McMorrow, D. F., Wilkins, S. B., Wastin, F., Colineau, E. & Fort, D. (2006). *Phys. Rev. Lett.* **97**, 137203. 10.1103/PhysRevLett.97.137203 17026068

[bb48] Walker, M. B., Buyers, W. J. L., Tun, Z., Que, W., Menovsky, A. A. & Garrett, J. D. (1993). *Phys. Rev. Lett.* **71**, 2630–2633. 10.1103/PhysRevLett.71.2630 10054730

[bb66] Watson, G. M., Gibbs, D., Lander, G. H., Gaulin, B. D., Berman, L. E., Matzke, H. & Ellis, W. (1996). *Phys. Rev. Lett.* **77**, 751–754. 10.1103/PhysRevLett.77.751 10062893

[bb67] Watson, G. M., Gibbs, D., Lander, G. H., Gaulin, B. D., Berman, L. E., Matzke, H. & Ellis, W. (2000). *Phys. Rev. B*, **61**, 8966–8975. 10.1103/PhysRevLett.77.751 10062893

[bb98] Wilhelm, F., Eloirdi, R., Rusz, J., Springell, R., Colineau, E., Griveau, J.-C., Oppeneer, P. M., Caciuffo, R., Rogalev, A. & Lander, G. H. (2013). *Phys. Rev. B*, **88**, 024424. 10.1103/PhysRevLett.114.097203 25793847

[bb35] Wilkins, S. B., Caciuffo, R., Detlefs, C., Rebizant, J., Colineau, E., Wastin, F. & Lander, G. H. (2006). *Phys. Rev. B*, **73**, 060406. 10.1103/PhysRevLett.100.076403 18352577

[bb38] Wilkins, S. B., Paixão, J. A., Caciuffo, R., Javorský, P., Wastin, F., Rebizant, J., Detlefs, C., Bernhoeft, N., Santini, P. & Lander, G. H. (2004). *Phys. Rev. B*, **70**, 214402. 10.1103/PhysRevLett.89.187202 12398632

[bb119] Willers, T., Strigari, F., Hiraoka, N., Cai, Y. Q., Haverkort, M. W., Tsuei, K.-D., Liao, Y. F., Seiro, S., Geibel, C., Steglich, F., Tjeng, L. H. & Severing, A. (2012). *Phys. Rev. Lett.* **109**, 046401. 10.1103/PhysRevLett.109.046401 23006099

[bb148] Wong, J., Krisch, M., Farber, D. L., Occelli, F., Schwartz, A. J., Chiang, T.-C., Wall, M., Boro, C. & Xu, R. (2003). *Science*, **301**, 1078–1080. 10.1126/science.1087179 12934002

[bb142] Wray, L. A., Denlinger, J., Huang, S.-W., He, H., Butch, N. P., Maple, M. B., Hussain, Z. & Chuang, Y. D. (2015). *Phys. Rev. Lett.* **114**, 236401. 10.1103/PhysRevLett.114.236401 26196808

[bb6] Zachariasen, W. H. & Ellinger, F. (1957). *J. Chem. Phys.* **27**, 811–812.

[bb5] Zachariasen, W. H. & Ellinger, F. H. (1955). *Acta Cryst.* **8**, 431–433.

[bb7] Zachariasen, W. H. & Ellinger, F. H. (1963*a*). *Acta Cryst.* **16**, 777–783.

[bb8] Zachariasen, W. H. & Ellinger, F. H. (1963*b*). *Acta Cryst.* **16**, 369–375.

[bb78] Zimina, A., Dardenne, K., Denecke, M. A., Doronkin, D. E., Huttel, E., Lichtenberg, H., Mangold, S., Pruessmann, T., Rothe, J., Spangenberg, T., Steininger, R., Vitova, T., Geckeis, H. & Grunwaldt, J. D. (2017). *Rev. Sci. Instrum.* **88**, 113113. 10.1063/1.4999928 29195371

